# Assessing the Impact of Whole Genome Duplication on Gene Expression and Regulation During Arachnid Development

**DOI:** 10.1093/gbe/evaf238

**Published:** 2025-12-05

**Authors:** Madeleine E Aase-Remedios, Daniel J Leite, Ralf Janssen, Alistair P McGregor

**Affiliations:** Department of Biosciences, Durham University, Durham DH1 3LE, UK; Department of Biosciences, Durham University, Durham DH1 3LE, UK; Department of Earth Sciences, Uppsala University, Uppsala 752 36, Sweden; Department of Biosciences, Durham University, Durham DH1 3LE, UK

**Keywords:** whole genome duplication, evolution, arachnids, spiders, gene regulation

## Abstract

Whole genome duplication (WGD) generates a new genetic material that can contribute to the evolution of developmental processes and phenotypic diversification. A WGD occurred in an ancestor of arachnopulmonates (spiders, scorpions, and their relatives), which provides an important independent comparison to WGDs in other animal lineages. After WGD, arachnopulmonates retained many duplicated copies (ohnologues) of developmental genes including clusters of homeobox genes, many of which have been inferred to have undergone subfunctionalization. However, there has been little systematic analysis of gene regulatory sequences and comparison of the expression of ohnologues versus their single-copy orthologues between arachnids. Here, we compare the regions of accessible chromatin and gene expression of ohnologues and single-copy genes during three embryonic stages between an arachnopulmonate arachnid, the spider *Parasteatoda tepidariorum*, and a nonarachnopulmonate arachnid, the harvestman *Phalangium opilio*. We found that the expression of each spider ohnologue was lower than their single-copy orthologues in the harvestman suggesting subfunctionalization. However, this was not reflected in a reduction in the number of peaks of accessible chromatin because both spider ohnologues and single-copy genes had more peaks than the orthologous harvestman genes. We also found that the number of peaks of accessible chromatin was higher in the late embryonic stage associated with activation of genes expressed later during embryogenesis in both species. Taken together, our study provides a genome-wide comparison of gene regulatory sequences and embryonic gene expression in arachnids and thus new insights into the impact of the arachnopulmonate WGD.

Significance The comparison of independent WGD events is essential to understand their evolutionary outcomes. Here, we examine gene expression and chromatin accessibility during the development of two arachnids, one of which underwent an ancient ancestral WGD. Our data provide the first direct comparison of the regulation of ohnologues and single-copy genes in arachnids.

## Introduction

**Fig. 1.**
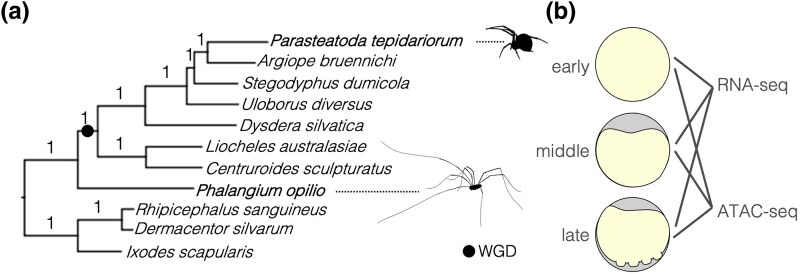
a) The *OrthoFinder* tree of OGs with one gene in all species for the 11 arachnid proteomes used in this study. The two focal species, *P. tepidariorum* and *P. opilio*, are in bold text. Branches are labeled with STAG support proportions. Silhouettes were taken from Phylopic.org, provided by Mathilde Cordellier and Gareth Monger under license: https://creativecommons.org/licenses/by-nc/3.0/. b) Schematic of early, middle, and late stages used for ATAC- and RNA-sequencing, corresponding to *P. tepidariorum* and *P. opilio* stages 5, 7, and 8 (Mittmann and Wolff 2012; Gainett et al. 2022).

Whole genome duplication (WGD) generates a new genetic material that can contribute to evolutionary diversification (Sémon and Wolfe 2007; MacKintosh and Ferrier 2017; Van de Peer et al. 2017). WGDs have occurred numerous times during animal evolution including the two rounds (2R) on the vertebrate stem and additional rounds in fish lineages, three in horseshoe crabs and a single event in the ancestor of arachnopulmonates, a lineage of arachnid arthropods including spiders, scorpions, and their relatives (Putnam et al. 2008; Sato and Nishida 2010; Kenny et al. 2016; Schwager et al. 2017; Nong et al. 2021; Kulkarni et al. 2025; Marlétaz et al. 2024) (Fig. 1). While the WGDs in vertebrates have been intensively studied, fully understanding the consequences of WGDs in animals requires comparison between these and the independent events in invertebrates.

Previous work has shown that the arachnopulmonate WGD resulted in spiders, scorpions, pseudoscorpions, amblypygids, and uropygids having retained duplicates (ohnologues) of many key developmental genes (Schwager et al. 2017; Akiyama-Oda and Oda 2020; Aase-Remedios et al. 2023; Kulkarni et al. 2025). For example, arachnopulmonates have two copies of many homeobox genes (including two Hox gene clusters), Sox genes, micro-RNAs, retinal determination genes, and Wnt-, Hedgehog- and Notch-Delta-signaling components, compared to other arachnid lineages that did not experience this event, such as harvestmen, which usually have only one copy (Fig. 1) (Schwager et al. 2007, 2017; Leite et al. 2016, 2018; Akiyama-Oda and Oda 2020; Baudouin-Gonzalez et al. 2021, 2024; Janssen et al. 2021; Aase-Remedios et al. 2023; Kulkarni et al. 2025). Many of these same genes were also retained in duplicate in vertebrates relative to unduplicated chordate outgroups such as amphioxus or tunicates (Dehal 2002; Mazet and Shimeld 2002; Cañestro et al. 2003; Woolfe and Elgar 2007; Putnam et al. 2008; Holland 2013).

Developmental genes involved in transcriptional regulation and signaling make up a larger proportion of ohnologues than expected by chance in vertebrates (Mazet and Shimeld 2002; Putnam et al. 2008; Singh et al. 2015). The sub- and neo-functionalization of these ohnologues may constitute the mechanism by which WGDs can facilitate the diversification of developmental processes and potentially evolutionary innovations (Cañestro et al. 2013; Mottes et al. 2021). The diversity of outcomes for ohnologues has given rise to several hypotheses. Firstly, ohnologues are initially redundant, so they may return to single copy if mutations result in the nonfunctionalization of one. Alternatively, one may acquire a new function so that the ohnologues are no longer redundant and both are retained (neo-functionalization), or the ohnologues may undergo subfunctionalization. The classical model of subfunctionalization by complementary degenerating mutations suggests that the loss of regulatory elements among ohnologues coincides with the loss of functions, usually expression domains (Force et al. 1999). The ancestral functionality is partitioned among the ohnologues, either with some redundancy, or in the case of dosage sensitivity, expression is initially reduced so that the ancestral unduplicated level of functionality is maintained, and retained ohnologues then undergo functional divergence (Edger and Pires 2009). It is likely that dosage sensitivity causes the initial preservation of ohnologues immediately following the WGD, while sub- and/or neo-functionalization occurs later as mutations accrue over time. Comparisons of gene expression and chromatin profiling between 2R vertebrates and the preduplicate state represented by the invertebrate chordate amphioxus revealed widespread subfunctionalization due to reduced regulatory complexity, as well as a new outcome, specialization (Marlétaz et al. 2018). Specialization was observed for a subset of genes that were expressed in most or all tissues in amphioxus. For these genes, one ohnologue was expressed in a highly restricted pattern relative to the ancestral gene but had gained, not lost, cis-regulatory elements consistent with a fine-tuning of regulatory complexity (Marlétaz et al. 2018). Both these mechanisms may contribute to the macroevolutionary consequences of WGD; however, regulatory data from lineages besides vertebrates is scarce. We have no knowledge of how the arachnopulmonate WGD has impacted gene regulation during spider development, although there is evidence of subfunctionalization of some developmental genes, including homeobox genes, during spider embryogenesis (Janssen et al. 2015; Leite et al. 2018; Baudouin-Gonzalez et al. 2021; Aase-Remedios et al. 2023; Janssen and Pechmann 2023).

To investigate this further, we carried out RNA-sequencing and characterized accessible chromatin using Assay for Transposase-Accessible Chromatin using sequencing (ATAC-seq) in the spider *Parasteatoda tepidariorum* and the harvestman *Phalangium opilio* (Fig. 1). For each species, we investigated three developmental stages that are morphologically comparable between the two species encompassing the formation of the germ band and the onset of segmentation when many key developmental genes are expressed and patterning is initiated by the Hox genes (Mittmann and Wolff 2012; Gainett et al. 2022). The early stage, stage 5, (Fig. 1b) sees the start of formation of the germ band and is defined in the spider *P. tepidariorum* by the migration of the cumulus to the rim of the germ disk to specify the dorso-ventral axis and the transition from radial to axial symmetry. The middle stage, stage 7, is characterized by the initiation of posterior segmentation and the formation of the first opisthosomal segments. The late stage, stage 8, is characterized by the onset of limb development, though this occurs earlier in harvestmen than spiders relative to the number of opisthosomal segments formed. These three stages were chosen with a central focus on the onset of posterior segmentation, and the distinct phases in development before and after, when many key developmental genes are expressed and patterning is initiated by the Hox genes. We also sequenced a contiguous genome assembly for the harvestman to serve as an improved reference for our data and better enable comparisons among arachnids, as the Arachnopulmonata–Opiliones ancestor is more recent than the ancestor between arachnopulmonates and the other arachnids with available reference genomes such as ticks or mites (Fig. 1a) (Ballesteros et al. 2022).

With these data, we aimed to understand how the arachnopulmonate WGD has impacted the gene content, genome structure, and chromatin accessibility and gene expression during embryogenesis in spiders. To do so, we first defined two sets of orthologous genes: one group with gene families containing one harvestman gene and two spider ohnologues and the other with gene families with one harvestman gene and one spider orthologue. We examined the expression levels as well as accessible chromatin between gene types (ohnologue or single copy) and species and among the stages. We also compared aspects of gene and genome structure including intron length and intergenic distance, as these parameters represent larger scale patterns of genome size evolution. Our data allowed us to examine temporal variation in expression, and we incorporated analysis of a previously published single-cell dataset for some late-stage-specific ohnologues we identified, allowing us to examine their spatial expression and, therefore, infer both temporal and spatial subfunctionalizations. We also used our accessible chromatin data to examine two examples of micro-synteny, comprising a newly described three-gene locus conserved between the harvestman and spider and the previously described homeobox gene clusters. Our updated genome assembly for the harvestman has greatly expanded our capabilities for syntenic comparisons focusing on the arachnopulmonate WGD. These data provide the first glimpse into the impact of the arachnopulmonate WGD on cis-regulatory sequences across the genome and gene expression at key developmental stages.

## Results

### Ohnologue Definition

To compare gene expression and regulation within and between species with and without an ancestral WGD, we first defined sets of ohnologues and single-copy genes using 11 available arachnid proteomes (Fig. 1a). To define the ohnologue set, we filtered orthogroups (OGs) defined by *OrthoFinder* (see Methods) to those with an average number of genes among WGD species between 1.5 and 2.5, an average number of genes between 0.5 and 1.5 among non-WGD species, where the two spider genes in the OG had to be on different chromosomes in at least two out of the three spiders with chromosome-level assemblies in our analysis (*P. tepidariorum*, *Argiope bruennichi*, and *Dysdera silvatica*), and those with one copy in *P. opilio* and two copies in *P. tepidariorum*. This conservative approach did not attempt to systematically define all ohnologues retained after WGD; we removed any ohnologue gene families with segmental duplications or gene losses in either species, resulting in 530 conservative 1:2 ohnologous gene sets. Single-copy orthologues were defined by *OrthoFinder* as the OGs containing a single gene across all 11 species (*n* = 1,435). Within ohnologous OGs, we defined the A ohnologue as the higher expressed ohnologue, and the B as the lower, unless ohnologues had been previously designated A or B in publications.

### Expression and Chromatin Dynamics Across Embryonic Stages

**Fig. 2.**
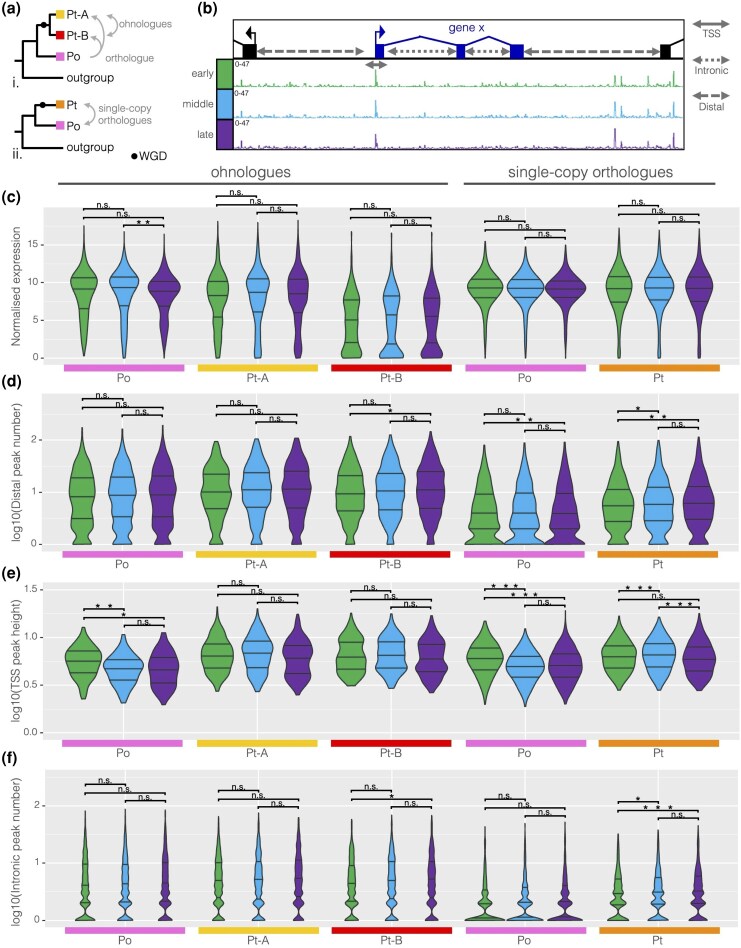
Expression and peak dynamics between stages for ohnologue and single-copy orthologue gene sets. a) Schematic illustrating the relationships between harvestman and spider genes in the case i. where both ohnologues are retained or ii. where one is lost and the spider retains a single orthologue. b) Schematic defining the intronic, distal, and TSS regions assigned to each gene with example ATAC profiles for each stage. c to f) Violin plots of normalized reads, number of peaks in the distal region, height of peaks overlapping with the TSS, and number of peaks within the introns for harvestman and spider ohnologue and single-copy orthologue gene sets. Significance levels were determined with a two-tailed Wilcoxon rank sum test (***P < 0.001; **P < 0.01; *P < 0.05; n.s. *P* > 0.05).

We first compared the expression and chromatin accessibility among ohnologue and single-copy orthologue gene sets between the three developmental stages of the spider *P. tepidariorum* and the harvestman *P. opilio* (Fig. 2a). We found that expression levels were largely consistent between stages for both ohnologue and single-copy orthologue gene families, except for a slightly significant decrease in expression of harvestman orthologues of spider ohnologues between early and late stages (Fig. 2c). This suggests that generally these sets of genes are employed and expressed at similar levels across all three stages of embryogenesis.

To compare the cis-regulatory regions of these genes, we then examined the number of distal and intronic peaks of accessible chromatin as well as peaks annotated proximally to the annotated transcription start site (TSS peaks) (Fig. 2b). We expected the number of distal and intronic peaks would increase, reflecting the gradual opening of chromatin at more enhancers during development. Consistent with this, for distal peaks, we observed significant increases in number between early and late stages in the spider B ohnologues, and both species' single-copy orthologues, and a significant increase between early and middle stages for spider single-copy genes (Fig. 2d; Table S1). The number of intronic peaks increased in time, in a similar pattern to distal peaks (Fig. 2f; Table S1).

As the genes in these sets are generally expressed at all three embryonic stages assayed and promoters are often constitutively open, we expected TSS peaks to remain constant throughout developmental time. However, TSS peak heights decreased in time, with significant decreases between early and late and/or early and middle stages for harvestman orthologues of spider ohnologues and both species' single-copy genes (Fig. 2e; Table S1). Mean TSS peak heights were lower for both spider ohnologues later compared to early stages, but this difference was not significant (Fig. 2e; Table S1). To confirm this pattern, we investigated whether there might be some genes with no TSS peaks in early or middle stages, meaning they would be excluded from the dataset when the logarithm was taken (i.e. log10 of 0 would return “-Inf”) resulting in them only being counted in late stages and decreasing the median or mean of the late-stage data; however, this did not affect the negative relationship of TSS peak height with time. Genes without TSS peaks may have misannotated start sites, resulting in no TSS peaks at any stage, rather than no TSS peak at any specific stage.

### Differences Between Species as Well as Ohnologue and Single-Copy Orthologue Gene Sets

**Fig. 3.**
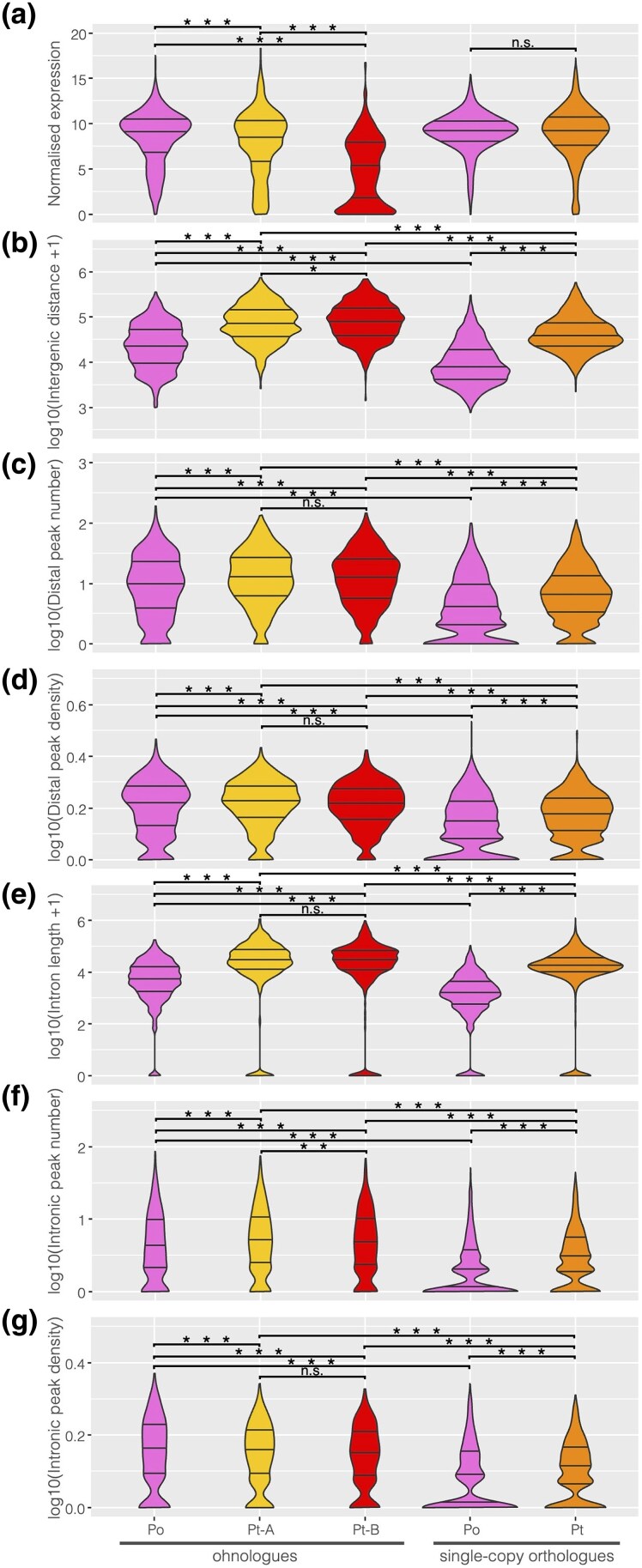
Differences in expression, ATAC peaks, intergenic distances, and intron lengths between harvestman and spider genes and between ohnologues and single-copy orthologues. a to g) Violin plots of normalized expression counts, intergenic distance, distal peak number and density, intron lengths, and intronic peak number and density per gene, for ohnologue and single-copy orthologue gene sets. Significance levels were determined with a two-tailed Wilcoxon rank sum test (***P < 0.001; **P < 0.01; *P < 0.05; n.s. *P* > 0.05).

While differences in ATAC peaks and expression between the stages were small and rarely significant, there were clear differences in these metrics between the species and between gene types when stages were combined. Under our null hypothesis, consistent with the outcome of strict dosage-balanced subfunctionalization, we predicted that both spider ohnologues would exhibit a decrease in expression to the same or a similar extent and that the sum of counts for spider A and B ohnologues would be nearly the same as their single-copy harvestman orthologues of spider ohnologues. Consequently, we also predicted that ohnologue expression would be lower than the spider single-copy genes, which we would predict to be equivalent to their harvestman orthologues (and to the harvestman orthologues of spider ohnologues). However, we found that the mean of normalized counts for ohnologue A, which we defined as the higher expressed ohnologue of the pair, was 7% lower than that of the harvestman orthologue, while ohnologue B, the lower expressed ohnologue, was 43% lower, so while both ohnologues were indeed lower in expression compared to the harvestman gene, ohnologue B was usually much lower (Fig. 3a; Fig. S2 and Table S1). As expected, we observed that the expression was not significantly different between the species' single-copy orthologues (Fig. 3a; Fig. S2).

We also noticed differences in gene structure and peak density between the two species, as well as between ohnologues and single-copy orthologues (Fig. 3). Firstly, spider genes have longer intergenic distances, more distal peaks, and longer introns than harvestman genes, regardless of belonging to ohnologue or single-copy orthologue gene sets (Fig. 3b, c, and e; Table S1). Since the *P. tepidariorum* genome (1009 Mb) is larger than that of *P. opilio* (476 Mb), this likely reflects differences in genome size and organization.

Consistent with this, spider ohnologues were found to have more intronic peaks than their harvestman orthologues, and spider single-copy genes had more intronic peaks than harvestman single-copy orthologues, though harvestman orthologues of spider ohnologues had more intronic peaks than spider single-copy genes (Fig. 3f). This pattern indicates differences between gene types besides species-specific differences.

Ohnologues had longer intergenic distances and intron lengths compared to single-copy genes, as did harvestman orthologues of ohnologues relative to harvestman single-copy orthologues (Fig. 3b and e). To investigate this further, we looked at the density of peaks in intergenic and intronic regions. Distal peak density was higher in ohnologues than single-copy genes (Fig. 3d; Table S1) and was similar between spider ohnologues and their harvestman orthologues. Peak density both reflects the higher number of distal peaks observed for spider ohnologues (Fig. 3c) and accounts for the smaller intergenic distances in the harvestman (Fig. 3b). A similar pattern was observed for intronic peaks; accounting for differences in intron length between species revealed differences in intronic peak number between the gene types (Fig. 3g). Ohnologues have higher intronic peak density, with no difference between A and B ohnologues. In the harvestman, orthologues of spider ohnologues have the highest intronic peak density, while single-copy orthologues had the lowest intronic peak density (Fig. 3g).

### Temporal Subfunctionalization

**Fig. 4.**
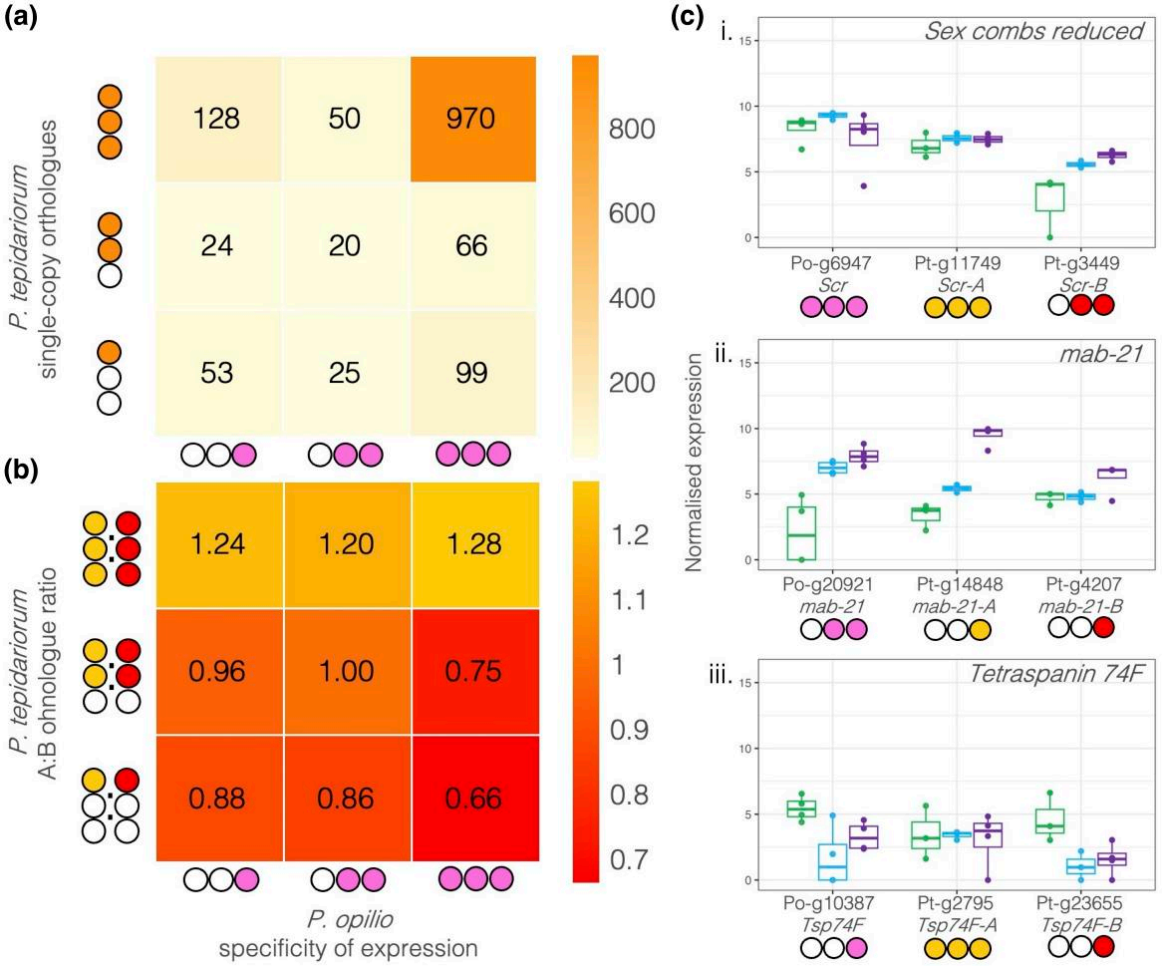
Stage specificity of expression of single-copy orthologues and spider ohnologues. a) Heatmap of the number of spider single-copy orthologues expressed in either a single stage, two, or three stages, by the number of stages their harvestman orthologues are expressed in. Stage specificity is denoted by the number of filled circles: expression in one, two, or all three stages. b) Heatmap of the ratio of spider A to B ohnologues expressed in either a single stage, two, or three stages, by the number of stages their harvestman orthologues are expressed in. c) Expression of examples of three ohnologue OGs with different stage specificities for the harvestman and spider genes. i. *Scr*, ii. *mab-21*, iii. *Tsp74F*. Gene names reflect previous annotations (Schwager et al. 2017) or annotated *P. tepidariorum* gene names on NCBI.

To further explore the temporal expression of genes in these two sets, we examined the number of stages each single-copy gene or ohnologue was expressed in relative to the number of stages its harvestman orthologue was expressed in using the relative expression (log2 fold change) between the stages. Most single-copy orthologues were expressed consistently across all three stages in both *P. tepidariorum* and *P. opilio* (Fig. 4a). We hypothesized that specialization would be evident for the spider ohnologues with respect to harvestman orthologues that were expressed across all three stages, similar to the nonspecific expression of amphioxus genes whose vertebrate ohnologues underwent specialization (Marlétaz et al. 2018). Consistent with this, we found that the lower expressed ohnologue B was more likely to be one- or two-stage specific, while the higher expressed ohnologue A was more likely to be expressed in all three stages (Fig. 4b). Furthermore, the difference in stage specificity between A and B ohnologues was highest for those with harvestman orthologues expressed in all three stages (Fig. 4b). This indicates that the lower expression of the B ohnologue is accordant with a temporal restriction in expression, rather than a uniform reduction in expression levels across all stages. This is particularly evident for the *Sex combs reduced* (*Scr*) genes (Fig. 4c.i), where *Pt-Scr-A* is expressed uniformly across all three stages, similar to *Po-Scr*, while *Pt-Scr-B* is expressed at similar levels to *Pt-Scr-A* at only the middle and late stages. Thus, the decrease in expression for *Pt-Scr-B* occurs by temporal restriction to middle and late stages. When both A and B ohnologues are single-stage specific in expression, the B ohnologue is expressed at a lower level, e.g. for *mab21* genes (Fig. 4c.ii). Comparing spider *Tetraspanin 74F* (*Tsp74F*) ohnologues to *Po-Tsp74F* shows that while *Pt-Tsp74F-A* has a higher average expression across the stages, *Pt-Tsp74F-B* has the highest expression of either ohnologue, but only in the early stage (Fig. 4c.iii), showing that stage specificity may be inversely related to the expression level. Overall, this shows that single-copy genes do not show differences in the specificity of their expression levels between the developmental stages we investigated, while ohnologues have different temporal expression patterns between stages, consistent with specialization or subfunctionalization of ohnologues.

### Ohnologue and Single-Copy Genes Have Different Annotated Functions

Based on the differences in chromatin accessibility and gene structure between the gene types, we then assessed the function of genes in ohnologue versus single-copy orthologue gene families. Gene Ontology (GO) biological processes for development, morphogenesis, and differentiation were overrepresented among ohnologues, while housekeeping functions such as metabolism, transport, synthesis, and translation were overrepresented among single-copy orthologue families (Fig. S3a). While single-copy genes were enriched for the GO biological process of “regulation of transcription by RNA-polymerase II,” the more specific term of “regulation of gene expression” was among the enriched terms for ohnologue families. Similarly, among terms related to protein synthesis, most were associated with single-copy genes, but proteins annotated with phosphorylation activities were overrepresented among ohnologues, suggesting ohnologues have functions as both transcription factors and kinases, potentially as components of signaling pathways. We also verified that GO terms were consistent between harvestman and spider reference genome annotations. As expected, GO terms were highly correlated between the species among ohnologue and single-copy gene sets (Fig. S3c.i).

### Early- and Late-Stage-Specifically Expressed Genes Have Different Annotated Functions

We also identified genes that were early or late specific in their expression patterns. The 352 harvestman and 521 spider genes with early-specific expression profiles were overrepresented for biosynthetic processes and intracellular localization, consistent with the functions in early development. The 276 harvestman and 434 spider late-specific genes were annotated almost exclusively for GO biological processes and GO molecular functions not found among early-specific genes, including cell adhesion, DNA binding, and Wnt signaling (Fig. S3b). We found again that GO annotations were highly correlated within the stage specificity groups and between species, confirming congruence in GO annotation between the two genomes (Fig. S3c.ii).

### Spatial Expression of Late-Expressed Genes

**Fig. 5.**
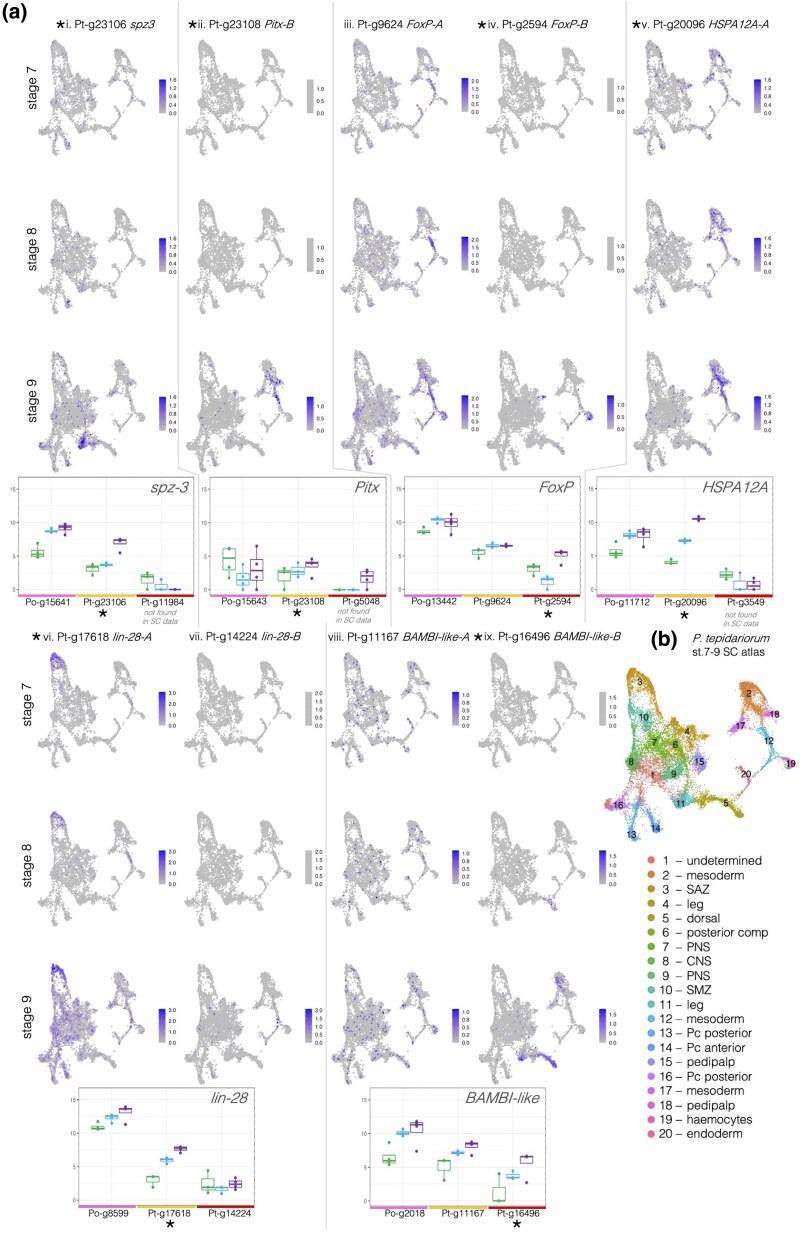
a) Expression of late-specific spider genes, their ohnologues, and harvestman orthologues in our data and the single-cell atlas from Leite et al. (2024). Genes that were late specific in their expression and had late-specific peaks are marked with asterisks. Gene names are based on the annotated spider name on NCBI or the closest fly orthologue, except for *Pitx-A* and *Pitx-B*, which were previously identified in Aase-Remedios et al. (2023), and *FoxP-A* and *FoxP-B*, which were previously identified in Janssen et al. (2022). b) Cell clustering and annotation as in Leite et al. (2024).

We were particularly interested in the spider genes that were late specific in their expression pattern and associated with an increase in the number of ATAC peaks annotated nearby, which we inferred to potentially represent the opening of chromatin at these genes' enhancers. We used differential peak numbers from comparisons between the three stages to determine if a gene was annotated with more peaks in the late stage relative to the early and middle stages. We found for most of the spider genes with late-specific expression and peaks that the harvestman orthologue was also expressed most highly in the late stage, for example, the Toll ligand *Po-spz3* (the harvestman orthologue of fly *spatzle 3*), *Po-HSPA12A* (the harvestman orthologue of *Heat shock 70 kDa protein 12A* that seems to lack a fly orthologue (Wada et al. 2006)), *Po-lin-28* (the harvestman orthologue of fly *lin-28*), and *Po-BAMBI-like* (the harvestman gene that seems to lack a fly orthologue but is similar to *BMP and activin membrane-bound inhibitor*); exceptions were *Po-Pitx* (the harvestman orthologue of *Pituitary homeobox*, orthologous to fly *Ptx1*) and *Po-FoxP* (the harvestman orthologue of fly *Forkhead box P*) (Fig. 5a.i to ix).

Given the development-related GO terms annotated to late-specific genes, we then used a previously published single-cell dataset from partially overlapping developmental stages (stages 7, 8, and 9, compared to our stages 5, 7, and 8) in *P. tepidariorum* (Leite et al. 2024) to determine whether spider genes that were late specific in expression were associated with any particular cell clusters. Unfortunately, many ohnologues were not found expressed in the single-cell dataset, hindering this large-scale investigation, so instead we focused on late-expressed genes with late-specific ATAC peaks to examine in more detail.

Of the 25 genes we identified with both late-specific expression and late-specific peaks, 20 belonged to ohnologue families. Some of these ohnologues were found to be present in specific cell clusters (Fig. 5b), namely, *Pt-spz3-A*, *Pt-Pitx-B*, *Pt-FoxP-B*, *Pt-HSPA12A-A*, *Pt-lin-28-A*, and *Pt-BAMBI-like-B* (Fig. 5.ii to vi and ix), though the rest were widely expressed and simply increased in expression levels over time (Fig. S4). *Pt-spz3-A* was detected in cells annotated to the leg cluster, while *Pt-spz-B* could not be identified in the single-cell data; our expression indicates it decreases in expression through time (Fig. 5a.i), so it may not be expressed during the stages that were assayed in the single-cell analysis. Low late-stage expression is likely also the reason that *Pt-HSPA12A-B* (Pt-g3549) was not detected in the single-cell data, while the late-specific *Pt-HSPA12A-A* was associated with cells annotated as the mesoderm (Fig. 5a.v). *Pt-Pitx-A* was also not detected in the single-cell data, though it was only detected in our late-stage RNA, which may indicate it is only lowly expressed. *Pitx-B* is found in cells annotated as the mesoderm. For the other late-specific ohnologue families with both copies found in the single-cell data, we could compare their expression to detect spatial subfunctionalization.

*Po-FoxP* and *Pt-FoxP-A* are expressed at similar levels in middle and late stages, and only *Pt-FoxP-B* is late-specific (Fig. 5a.iii to iv). Both spider *FoxP* ohnologues are expressed at lower levels relative to *Po-FoxP*, consistent with subfunctionalization. *FoxP* ohnologues are also found in different cell clusters, with *Pt-FoxP-A* expressed in cells annotated as the mesoderm (cluster 12) and central nervous system (CNS; cluster 8) (Fig. 5a.iii) and *Pt-FoxP-B* in cells annotated as hemocytes (cluster 19) and in the developing legs (cluster 4) (Fig. 5a.iv). This is largely consistent with published expression data where *Pt-FoxP-A* was detected in later stages (10+) in the CNS, with stage 8 to 9 head expression that could represent mesodermal cells in cluster 12, and *Pt-FoxP-B* was detected in hemocytes (*Pt-FoxP-B* is *FoxP1*; *Pt-FoxP-A* is *FoxP2*) (Janssen et al. 2022).

The differences between *Po-Pitx* expression and *Pt-Pitx-A* and *Pt-Pitx-B* appear to reflect both the subfunctionalization of the spider ohnologues and differences in the timing and expression patterns between the species, with *Po-Pitx* expressed highest in the early stage, lowest in the middle, and then slightly higher in the late stage, while both spider ohnologues are highest during the late stage (Fig. 5a.ii to iii). *Pt-Pitx-B* was detected in cells annotated as the mesoderm (clusters 12 and 2) and the pedipalps (cluster 18) (Fig. 5a.ii). *Pt-Pitx-A* was not detected in early- or middle-stage embryos, indicating it may be activated later than *Pitx-B*, and it was not detected in the single-cell data, suggesting it is only very lowly expressed until stage 9. This is largely consistent with expression data from later stages (stages 10 to 12) showing that both *Po-Pitx* and *Pt-Pitx-B* are expressed in the precheliceral region, while similar patterns to the expression of *Po-Pitx* along the ventral midline are partitioned between *Pt-Pitx-A* and *Pt-Pitx-B* (Leite et al. 2018). Note that we are using names as in Aase-Remedios et al. (2023), so, in this case, *Pitx-A* is the lower-expressed ohnologue; *Pitx-A* is *Pitx-1*, and *Pitx-B* is *Pitx-2* in Leite et al. (2018).

Expression differences consistent with spatial and temporal subfunctionalization were also seen for the other late-specific genes with retained ohnologues. *P. tepidariorum lin-28-A* is expressed in cells annotated as the segment addition zone (SAZ) at stages 7, 8, and 9, though expression was also detected across many other cell clusters at stage 9 (Fig. 5a.vi). *Pt-lin-28-A* is also expressed at a low level in the cells annotated as cluster 12 (mesoderm) throughout all these stages. Its ohnologue *Pt-lin-28-B* is not expressed at stages 7 and 8, and expression was at a much lower level at stage 9, with its highest expression in cluster 12, and it was detected in a few cells that also express *lin-28-A* (Fig. 5a.vii). *Pt-lin-28-B* may be expressed in later stages relative to *Pt-lin-28-A*, but it is clear that *lin-28-B* is expressed in fewer cells and primarily in different cell clusters relative to *Pt-lin-28-A*.

From these data, we were able to detect expression patterns consistent with spatial and temporal subfunctionalization, among newly identified late-expressed ohnologues, which with further investigation may reveal more about the impact of WGD on the spider development.

### Double-Conserved Synteny of a Late-Specific Locus

**Fig. 6.**
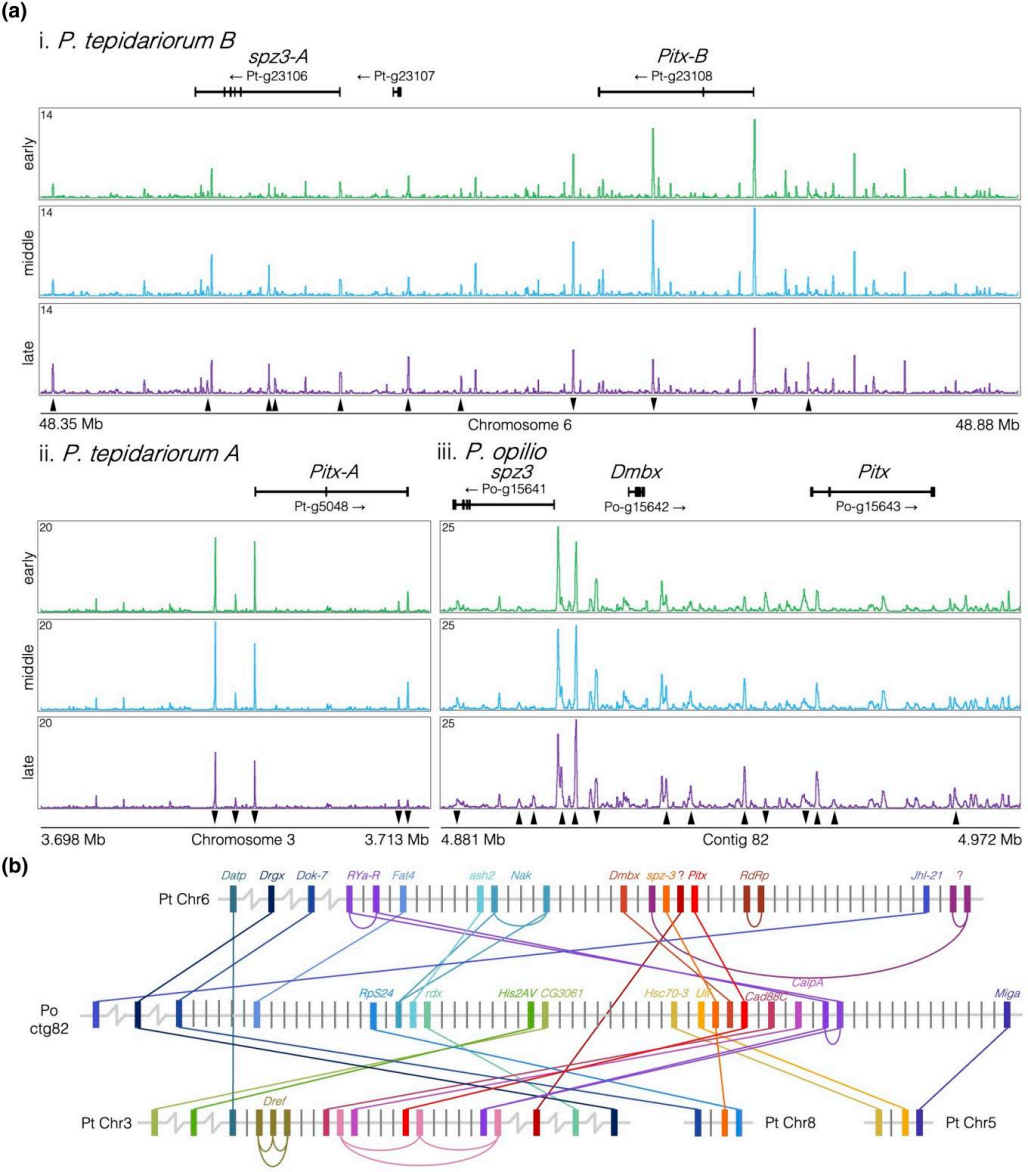
Chromatin profiles and synteny surrounding the conserved late-specific *spz3–Pitx* locus in *P. tepidariorum* and *P. opilio*. a) Chromatin accessibility profiles for i. the *Pt-spz3-A–Pt-Pitx-B* locus on chromosome 6 in the spider, ii. the *Pt-Pitx-A* locus on chromosome 3 in the spider, and iii. the *Po-spz3–Po-Pitx* locus on contig 82 in the harvestman. Solid arrowheads indicate the significant DiffBind change in peak height for peaks that are higher in the late stage (pointing up) or early stage (pointing down). b) Schematic of gene families with members linked to *Pitx* loci in the two species. Families are colored according to their relative position along the harvestman contig 82, and connected by lines indicating their relationships. Names represent previous annotations (Aase-Remedios et al. 2023) or the name of the fly orthologue and question marks indicate uncharacterized genes without an identified fly orthologue.

We noticed that two of the late-specific genes, *Pt-Pitx-B* and *Pt-spz3-A*, were located one gene apart, as were the harvestman orthologues of these genes, *Po-Pitx* and *Po-spz3*. This prompted us to identify the late-specific peaks in the conserved *spz3–Pitx* locus and investigate the broader synteny surrounding it (Fig. 6).

We identified several late-specific peaks near the promoter of *spz3* and one upstream of *Pitx-B*, which correlate with the late-specific expression of these genes in the spider (Figs. 6a.ii and 7a.i). Within the second intron of *Pitx-B*, we also identified a peak that was high at both early and middle stages but lower at the late stage (Fig. 6a.i), in a pattern directly inverse to *Pitx-B* expression (Fig. 5a.ii). In the ohnologous *Pitx-A* locus, we did not identify any late-specific peaks; rather, all peaks surrounding this gene were lower at the late stage relative to the early or middle stages (Fig. 6a.ii), inversely corresponding to the late-specific *Pitx-A* expression (Fig. 5a.ii). In the harvestman, we identified a peak proximal to the promoter of *Po-Pitx* that was highest in the early stage, lowest in the middle stage, and increased again from middle to late (Fig. 6a.iii), mirroring the expression pattern observed for *Po-Pitx* in our data (Fig. 5a.ii).

**Fig. 7.**
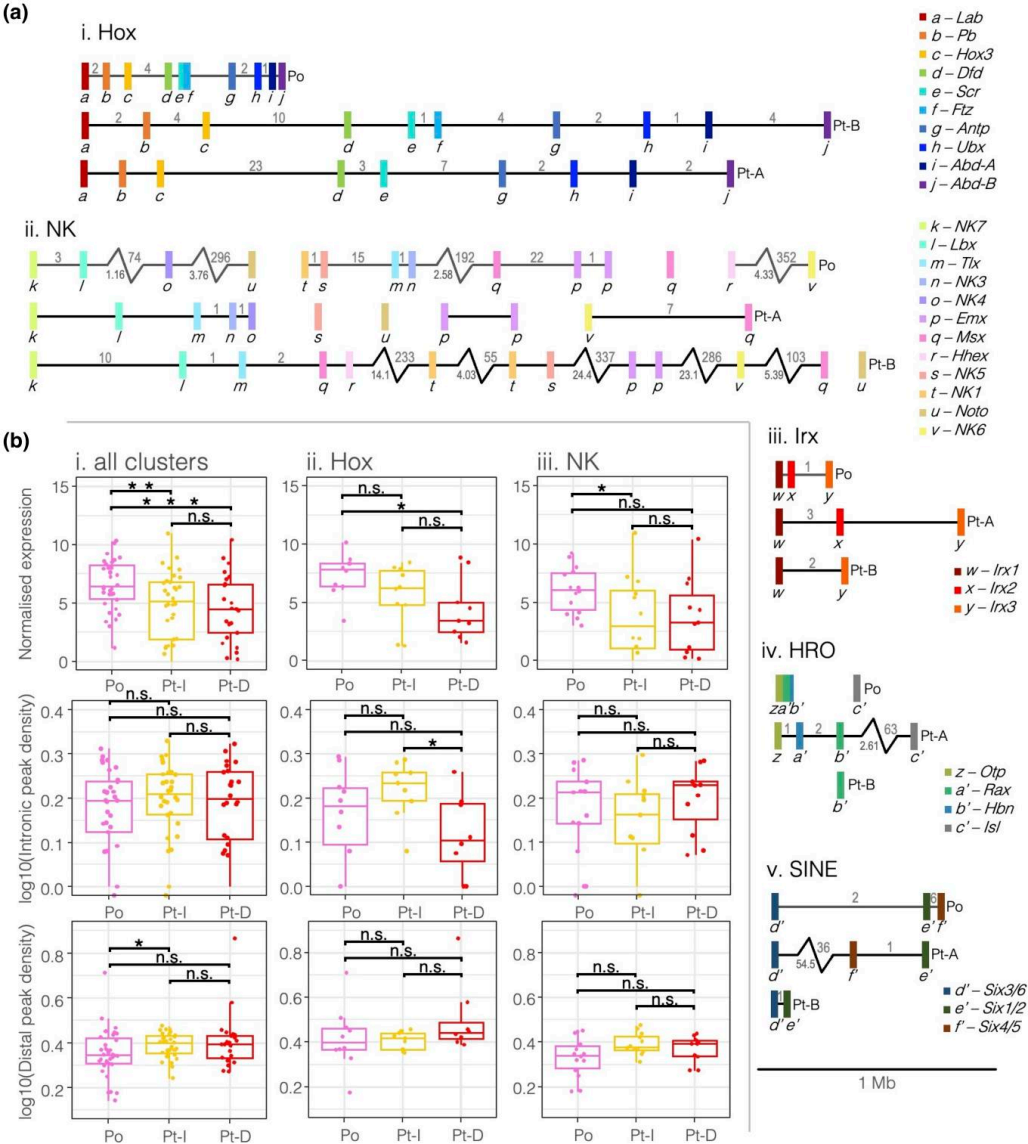
Homeobox gene organization, expression and ATAC peak density metrics in the spider and harvestman. a) To-scale schematic of conserved homeobox gene clusters in the spider and harvestman: i. Hox, ii. NK, iii. Irx, iv. HRO, and v. SINE. Black or gray lines represent chromosomes and contigs, respectively. Colored rectangles indicate homeobox genes, plotted by the position of the homeobox; letters below as well as the color scheme indicate the gene family, with the key to the right. Numbers above the chromosome/contig lines indicate the number of genes between each homeobox gene on either side. The zig-zag bar indicates a long distance, the length of which in Mb is denoted below in gray. The full annotation of *P. opilio* homeobox genes can be found in Supplementary File S2. b) Expression, intronic peak density, and distal peak density for i. all homeobox clusters depicted in a) and for ii. just Hox and iii. just NK genes. Pt-I refers to the more intact cluster (cluster A for all clusters besides Hox, as Hox-B is the more intact cluster, after Schwager et al. (2017)), and Pt-D refers to the more dispersed cluster (cluster B for all clusters besides the Hox).

Given the conservation of the two genes, we examined the organization of this locus in both species. The spider *Pt-spz3-A–Pt-Pitx-B* locus is nearly six times larger than the *Po-spz3–Po-Pitx* locus in the harvestman, comprising 0.53 Mb compared to 0.09 Mb, and *Pt-Pitx-B* is inverted relative to *Po-Pitx* (Fig. 6a.i and iii). The intervening gene in the harvestman is another homeobox gene, *Po-Dmbx* (*Diencephalon/mesencephalon homeobox*, no fly orthologue) (Fig. 6a.iii), the spider orthologue of which can be found five genes away (∼0.36 Mb) from *Pt-Pitx-B* on chromosome 6 (Fig. 6b). We searched for other homeobox gene families with a similar linkage pattern and found that *Drgx* (*Dorsal root ganglion homeobox*, orthologous to fly *Drgx*) genes are linked to *Po-Pitx* in the harvestman, as well as *Pt-Pitx-B* on chromosome 6 and *Pt-Pitx-A* on chromosome 3 in the spider.

We then searched for genes within 20 genes on either side of each *spz3–Pitx* locus in harvestman or spider that have orthologues or ohnologues linked to any of the other two *spz3–Pitx* loci. *Pt-spz3-B* is not linked to *Pt-Pitx-A*, but there are 12 gene families connecting any two of the three *spz3–Pitx* loci (the three loci comprising the contig bearing the harvestman *spz3–Pitx* locus and the two spider chromosomes containing *spz3-A–Pitx-B* or *Pitx-A*), while three families connect the harvestman *spz3–Pitx* locus to spider chromosome 5, and three families connect the harvestman *spz3–Pitx* locus to spider chromosome 8 (bearing *Pt-spz3-B*), indicating multiple interchromosomal rearrangements (Fig. 6b). All the arachnids surveyed in Aase-Remedios et al. (2023) also exhibit linkage between *Pitx*, *Drgx*, and *Dmbx* genes (Supplementary File S1) on two or more chromosomes in the spiders, indicating these genes were linked in the arachnid ancestor, and this linkage was conserved in two ohnologous loci following the WGD.

### Homeobox Gene Cluster Organization in the Harvestman *P. opilio*

We also examined the chromatin accessibility surrounding other homeobox genes in conserved clusters and subclusters. We previously characterized the homeobox gene complement of many arachnids, and we found that ancient homeobox gene clusters such as the Hox, NK, SINE, HRO, and Iroquois (Irx) clusters were retained with one more intact copy, while the second copy underwent varying degrees of dispersal via gene losses and rearrangements following the arachnopulmonate WGD (Aase-Remedios et al. 2023). Here, we classified the homeobox genes in our improved reference genome assembly for *P. opilio* with the aim of comparing the organization of homeobox gene clusters to the expression and chromatin accessibility between clusters with differing levels of organization (Fig. 7a). We hypothesized that intact homeobox clusters might have smaller intergenic distances, more peaks, or differences in expression compared to the more dispersed clusters. Expression is significantly lower for spider homeobox genes than for harvestman genes, but spider homeobox genes have higher intronic and distal peak densities (Fig. 7b.i).

We then compared only the Hox and NK clusters, two multi-gene ANTP-class clusters with different levels of conservation and organization (Aase-Remedios et al. 2023). The harvestman Hox cluster is compact and organized, while both spider Hox clusters are organized, but are larger and have more intervening genes, and even more so for the Hox-A cluster, which has lost *ftz*, making it the more dispersed of the two (for all other clusters, the B cluster is more dispersed) (Fig. 7a.i). The NK genes are organized to a similar extent in the harvestman and spider, comprising smaller subclusters of two or three NK genes separated by large distances, onto different contigs, or even chromosomes (Fig. 7c.ii). The harvestman NK cluster is found on four contigs, one with *NK7*, *Lbx*, *NK4*, and *Noto*, another with *NK1*, *NK5*, *Tlx*, *NK3*, *Msx1*, *Emx1*, and *Emx2*, a third with *Msx2*, and a fourth with *Hhex* and *NK6* (Fig. 7c.i). Some clustered gene pairs in the harvestman mirror the conserved clustering in the spider, indicating that subclustering of certain NK gene pairs may be constrained in both species. These conserved pairs consist of *Po-NK7–Po-Lbx* and *Pt-NK7-A–Pt-Lbx-A*, *Po-Tlx–Po-NK3* and *Pt-Tlx-A–Pt-NK3*, *Po-NK1–Po-NK5* and *Pt-NK1-2–Pt-NK5-B*, and *Po-Emx1–Po-Emx2* and both *Pt-Emx1-A–Pt-Emx2-A* and *Pt-Emx1-B–Pt-Emx2-B*.

Hox-A cluster expression was significantly lower than that of the genes in the harvestman Hox cluster, and lower than the expression of intact cluster genes in Hox-B, though this decrease was not significant (Fig. 7b.ii). This pattern mirrors the difference in expression between harvestman orthologues and spider ohnologues (Fig. 3a). For the NK cluster, the intact copy, which contains the more conserved “core” NK cluster (NK-A), was significantly lower expressed than the harvestman NK genes, while the more dispersed NK-B was also lower, but not significantly different from the harvestman (Fig. 7b.iii). The intact Hox-B cluster also had significantly higher intronic peak density than the dispersed Hox-A cluster and was not significantly higher than the harvestman Hox cluster intronic peak density (Fig. 7b.ii). There were no significant differences in peak density metrics between the NK clusters.

The other homeobox gene clusters do not contain enough genes to reveal any discernible patterns in expression or peak metrics. However, they have varying degrees of organization relative to the spider clusters (Fig. 7c). The harvestman Irx cluster is smaller than either of the spider clusters, and the HRO cluster of the harvestman is extremely compact relative to the spider. Both the harvestman HRO and SINE clusters are inverted relative to the spider clusters, though in which lineage this occurred cannot be determined. The spacing of *Six3/6* further away from *Six1/2* and *Six4/5* in the harvestman matches that seen in the intact *P. tepidariorum* SINE-A cluster, and the harvestman Irx cluster is similarly proportionally spaced to the *P. tepidariorum* Irx-A cluster as well. These organizations illustrate that the diversity of levels of organization observed between spider homeobox gene clusters are also present in the smaller *P. opilio* genome. To-scale figures of conserved core clusters and subclusters, including intervening genes, with chromatin profiles can be found in Supplementary File S3.

## Discussion

The data we present here adds new depth to our understanding of the impact of the arachnopulmonate WGD on gene expression during development. We found that spider ohnologues are expressed at lower levels relative to their harvestman orthologues, with one ohnologue typically being lower expressed than the other, indicating an asymmetrical outcome of subfunctionalization. Under the standard model of subfunctionalization, we expected a decrease in the number of peaks in the lower-expressed ohnologue consistent with the loss of regulatory elements (Force et al. 1999), while under specialization, we would expect an increase consistent with the fine-tuning of regulation (Marlétaz et al. 2018). However, we did not find any significant differences in the regulatory complexity (i.e. peak number or peak density) between ohnologues in the spider, despite an asymmetrical decrease in expression between ohnologues (Fig. 3). This suggests that neither subfunctionalization nor specialization is more prevalent than the other on a genomic scale; rather, these processes are likely acting on a gene-by-gene basis.

We also observed different dynamics for TSS peaks compared to distal and intronic peaks over time (Fig. 2). TSS peak heights decreased over time, and this difference was significant for all genes except spider ohnologues. Distal peaks and intronic peaks, however, increased significantly in number in the spider and showed a nonsignificant increase in the harvestman. Distal peaks are likely more dynamic, representing potential enhancers; thus, the increases in distal peak numbers may represent the gradual opening of chromatin at cis-regulatory elements as development proceeds. The decrease in TSS peak heights amid the opening of more distal and intronic peaks may reflect that over time more and more of the genome is in regions of open chromatin, causing a dilution of the Tn5 transposase relative to the amount of accessible genome sequence, resulting in shorter constitutive peaks (like those at promoters) due to the increase in the number of new peaks.

There were interesting differences between the two species and between ohnologues versus single-copy genes in how chromatin dynamics changed with time, suggesting these genes might be structurally different. When we examined intron size, intergenic distances, and intronic and distal peak number and density for combined stages, we found differences in regulatory complexity and expression between the gene families with retained ohnologues relative to those that returned to the single copy. While the spider has a larger genome, so we would predict longer introns and intergenic distances, we also saw that spider and harvestman genes in ohnologue gene families had longer introns and intergenic distances than those in single-copy gene families, but also had higher distal and intronic peak density than either species' genes in single-copy gene families. Therefore, the arachnopulmonate WGD seems to have impacted the genome structure of the spider beyond duplicate gene retention, resulting in longer introns and larger intergenic distances, which is also true of vertebrate genomes relative to outgroups where gene number does not increase proportionally with genome size (Ohno 1999). We can infer that the genes that were later retained in duplicate in the spider following WGD had higher regulatory complexity in the spider–harvestman ancestor, because harvestman orthologues of spider ohnologues have higher peak density than either the harvestman or spider single-copy orthologues. This indicates regulatory complexity may be a factor in determining a gene's propensity for retention in duplicate, as regulatory elements act as a substrate for mutations with functional effects.

Ohnologues were enriched for developmental functions, including regulation of transcription and signaling pathways, consistent with previous findings (Putnam et al. 2008; Berthelot et al. 2014). Our conservative definition of ohnologues to enable our two-species comparisons here identified 530 ohnologous gene families between *P. tepidariorum* and *P. opilio*, comprising 530 harvestman genes and 1,060 spider genes. This is likely an underestimation of the true number of retained spider ohnologues originating from the arachnopulmonate WGD and should not be interpreted as a count of all the ohnologues in *P. tepidariorum*. Relaxing our definitions to allow for independent tandem duplications or gene losses in the outgroup or the other spider species we used to define them, as well as rearrangements bringing ohnologues onto the same chromosome, would increase the number of detected ohnologue gene families.

We also compared early- and late-specifically expressed genes and found that late-specific genes were enriched for interesting developmental functional annotations. We then examined genes that were expressed specifically in the late-stage RNA data and had corresponding distal peaks that increased in the late-stage ATAC data, as these would be genes expressed during the formation of the germ band and posterior segmentation. Twenty of 25 of these late-specific genes had retained ohnologues and included several interesting developmental genes (Fig. 5 and Fig. S4). Two of the late-specific genes were found in a conserved arrangement in both the spider and harvestman genomes and revealed a pattern of double-conserved synteny resulting from the WGD (Fig. 6). In the harvestman, *Po-Pitx* was not late specific in expression, but *Po-spz3* was (Fig. 5a.i to ii). Gene linkage is thought to be constrained by shared regulatory elements (Irimia et al. 2012), but here, it appears that there has been an inversion in one of the lineages, which may have affected any coregulation between these genes. This could relate to how in the spider, *Pt-Pitx-B* and *Pt-spz3* are in the same orientation in the genome and are both late specific in expression, while the two genes are in the opposite orientation to one another in the harvestman and have different temporal expression patterns, though this remains to be tested.

What was also apparent from the conservation of this locus was a pattern of 1:2 synteny resultant from the arachnopulmonate WGD. Many genes linked to the harvestman *spz3* and *Pitx* genes could be found on chromosome 6 or chromosome 3 in the spider, which bear the two *Pitx* ohnologues. While there are many gene families with only 1:1 relationships among harvestman and spider *Pitx* loci, this pattern still illustrates the 1:2 conserved synteny resulting from the WGD, as single-copy orthologues are found linked to one or the other of the two *Pitx* ohnologues. WGD syntenic patterns can be obscured by gene losses or rearrangements. Our synteny analysis included gene families that underwent the loss of a harvestman orthologue [e.g. the paralogues *Pt-g23017* and *Pt-g5092* {uncharacterized family, adjacent to *Pt-Pitx-B* and linked to *Pt-Pitx-A*, respectively}] and a spider ohnologue (e.g. *Pt-Dmbx* and *Po-Dmbx*) as well as the rearrangement onto another chromosome, in this case (e.g. *Po-Uif* and *Pt-Uif* on chromosome 6) (Fig. 5b). This is consistent with the patchwork arrangement of vertebrate ohnologues across four paralogous chromosomes due to gene losses and rearrangements (McLysaght et al. 2002; Dehal and Boore 2005; Singh et al. 2015). This is also consistent with findings that the 1:2 pattern of synteny is present, but somewhat obscured in spider genomes due to extensive rearrangements, but are apparent among homeobox gene clusters (Fan et al. 2021; Miller et al. 2023; Aase-Remedios et al. 2023; Kulkarni et al. 2025).

With our improved genome assembly, we have now described the homeobox gene complement and organization in the harvestman (Fig. 7a; Supplementary File S2) and enabled better detection of the linkage between homeobox genes compared to previous assemblies (Gainett et al. 2021). We confirmed that there is a single intact Hox cluster containing all ten genes and found that the other homeobox clusters exhibit different levels of organization in *P. opilio*, with a very condensed HRO cluster and similar relative spacing of genes in the SINE and Irx clusters in spiders and the harvestman. The harvestman NK genes can be found in dispersed subclusters, some of which are conserved in two copies in the spider; though without chromosome-scale resolution, it is not clear if some of the NK subclusters are linked. We previously described the expression of NK cluster genes in the harvestman and found that harvestman *NK7*, *Lbx*, *Tlx*, and *NK3* are all expressed in the limbs, and the two *Emx* paralogues are expressed in nearly identical patterns (Leite et al. 2018; Aase-Remedios et al. 2023), which may implicate cis-regulatory elements in the coregulation and constraint on the organization of these subclustered genes. We were unable to identify any conserved noncoding sequence associated with any peaks in NK subclusters between the species due to their evolutionary divergence, so this remains to be investigated. The similarities between the organization of homeobox genes and the conserved synteny of the late-specific *spz3–Pitx* locus indicates that the genome of the harvestman may be very useful single-copy outgroup for future chromosome-scale syntenic comparisons, more so than other nonarachnopulmonate outgroups such as ticks or mites. However, further genome-wide comparisons of macrosynteny relative to ancestral linkage groups are necessary to determine the extent of syntenic conservation in the harvestman relative to other arachnids.

## Conclusion

Our analysis of regulatory regions and gene expression between equivalent embryonic stages of a spider and a harvestman provides evidence that the WGD has impacted the regulation of duplicated genes, many of which have roles in development. Specifically, we showed that ohnologues have likely undergone subfunctionalization, were characterized by higher regulatory complexity relative to single-copy genes, and were enriched for GO functions consistent with roles in development and signaling. This may reflect the propensity of developmental genes, which are thought to have more complex cis-regulation, to be retained in duplicate, and is consistent with observations in vertebrates. Using our new reference genome for the harvestman *P. opilio*, we were able to examine the synteny of homeobox genes and a late-specific locus, further providing evidence for the arachnopulmonate WGD. This resource improves upon available genomes from preduplicate arachnids to better understand the ancestral state giving rise to both Opiliones and arachnopulmonates and the impact of the arachnopulmonate WGD.

## Methods

### RNA-Seq and ATAC-Seq Protocols

We sampled embryos of the harvestman *P. opilio* collected from a wild population in Uppsala, Sweden, in October 2023. Due to somewhat nonsynchronous development, batches of embryos were dechorionated with 1:1 bleach:water solution and sorted into early germ band (stage 5), onset of posterior segmentation (stage 7), and limb-bud formation (stage 8) stages (Gainett et al. 2022) (Fig. 1b). Samples of 25, 15, and 10 embryos for each stage, respectively, were collected for ATAC protocols (Diagenode C01080002) to account for fewer cells in younger embryos, and the remaining embryos from each batch were used for RNA extraction with TRIzol in four replicates for each stage. The standard Diagenode protocol was carried out four times for each stage, with the additional step of using a hemocytometer to determine the amount of lysate containing 50,000 nuclei to use for tagmentation. We then sampled equivalent stages of embryos of *P. tepidariorum* from the lab culture in Durham, United Kingdom, for ATAC sequencing in four replicates for each stage and conducted the same protocol as above. We sampled three replicates for the early (stage 5) and middle (stage 7) stages and four replicates for the late (stage 8) stage of *P. tepidariorum* embryos for RNA extraction (Mittmann and Wolff 2012). ATAC libraries for the 12 samples of each species were pooled at equimolar concentrations for sequencing.

### High Molecular Weight DNA Extraction

We aimed to improve upon the reference genome assembly of the harvestman *P. opilio* using long-read sequencing. We sampled the anterior part of the body up to posterior of the fourth limb-bearing segment and including the limbs of a single female *P. opilio* caught from the wild population in Uppsala, Sweden. We extracted high molecular weight (HMW) DNA with the Monarch kit (T3060S), but instead of using the beads to precipitate DNA, we used a centrifuge (5000 rpm, 15 min) to pellet the DNA after the addition of isopropanol and then continued with the washing steps.

### Quality Control and Sequencing

ATAC libraries were quality checked on the Agilent TapeStation with a D1000 high-sensitivity assay. The HMW DNA extraction concentration was measured with a Qubit spectrophotometer and quality checked on the Agilent TapeStation with the Genomic DNA assay. ATAC libraries were paired-end sequenced (150 bp) on an Illumina NovaSeq by Novogene, yielding 2.32 B (407 Gb) and 2.71 B (347 Gb) reads for *P. opilio* and *P. tepidariorum*, respectively. cDNA libraries were prepared from RNA extractions with poly-A enrichment and paired-end sequenced with 150 bp fragments as well, yielding a total of 1.18 B reads (177.3 Gb) for both species, with an average of 54 million reads per sample. Libraries for the HMW DNA were prepared for PacBio Revio Hi-Fi sequencing by Novogene, yielding 8.2 M reads (101.11 Gb).

### Draft Harvestman Genome Assembly

Adapters were removed from the HiFi reads with *HiFiAdapterFilt* (Sim et al. 2022), then the mitogenome was assembled with *MitoHiFi v2.2* (Uliano-Silva et al. 2023), and reads not mapping to it were isolated with *minimap2 v2.24* (Li 2021). These nonmitochondrial reads were assembled with *hifiasm v0.19.9* with the *s* parameter set to 0.2 for higher heterozygosity due to the sample originating from a wild population (Cheng et al. 2021), and the assembly was reduced to a single pseudohaplotype with *purge_dups v1.2.6* (Guan et al. 2020). The assembly was then aligned with *blastn* (Altschul et al. 1990) to the *Genbank Refseq* RNA database (https://ftp.ncbi.nlm.nih.gov/blast/db/refseq_rna.14.tar.gz) to identify contaminants. Read coverage was determined with *minimap2* (Li 2021), and completeness was calculated with *BUSCO v5.5.0* using the *arachnida_odb10* reference (Manni et al. 2021). These genome statistics were visualized with *BlobTools2*, and the assembly was filtered to remove sequences with GC proportions below 0.35 or above 0.45, coverage lower than 20, length shorter than 10 kb, and hits from nonanimal kingdoms (Challis et al. 2020). The final assembly comprises 649 Mb across 123 contigs, with an N50 of 9.88 Mb and a BUSCO completeness score of 93.6%. Repeats were identified with *RepeatModeler* with *LRTStruct* and *quick* options and masked with *RepeatMasker* with *xsmall*, *gff*, and *rmblast* options (Smit and Hubley 2023). Assembly statistics can be found in Fig. S1. Raw HiFi reads were deposited in GenBank with the identifier SRR31693326 under BioProject PRJNA1197178. This final assembly was deposited in GenBank under the accession JBJXVG000000000. The version described in this paper is JBJXVG010000000.

### RNA-Seq Analysis

To compare RNA expression between *P. opilio* and *P. tepidariorum*, we generated gene annotations using RNA-seq evidence for in silico prediction with *BRAKER3* (Gabriel et al. 2024). The RNA-seq data generated in this study for *P. opilio* and RNA-sequencing reads from pooled developmental stages of *P. tepidariorum* (GenBank: SRR1507193 and SRR1507194) were trimmed of adapters and low-quality bases using *cutadapt v4.1* (Martin 2011). Trimmed reads were aligned to the reference genome of *P. tepidariorum* (Zhu et al. 2023) with our annotation or the de novo assembly and annotation for *P. opilio* with *STAR v2.7.9a* (Dobin et al. 2013). We used *DESeq2* in *R v4.3.1* for differential gene expression analysis (Love et al. 2014). To enable comparisons between sequencing runs and between species, we standardized the number of reads across samples and between species, relative to the total number of reads mapped to genes per sample per species. While DESeq2 accounts for variation in library size within matrices, to better enable comparisons between species, the read counts for each sample for each species were scaled relative to the sample with the largest number of mapped reads out of the two species, though read count matrices were kept distinct for the two species for differential gene expression analysis and were normalized using the *normTransform*() function from *DESeq2* for plotting read counts between species. Raw RNA-seq reads were deposited in GenBank under the BioProject PRJNA1198402.

### ATAC-Seq Analysis

To compare chromatin accessibility between *P. opilio* and *P. tepidariorum*, we called ATAC-seq peaks for our three developmental stages. Adapters were removed with *cutadapt v4.1*, and reads were aligned to the reference genomes with *Bowtie2 v2.4.5* (Langmead and Salzberg 2012). Using *SAMtools v1.17* (Danecek et al. 2021), alignments were filtered and sorted, duplicate reads were removed, and replicate runs of samples were merged. We recentered reads as previously (Buenrostro et al. 2015) and calculated effective genome size with *khmer v2.1.2* (Crusoe et al. 2015) before peak calling with *MACS2 v2.2.7.1* (Zhang et al. 2008). We used *DiffBind* in *R v4.3.1* to determine significant differences in peaks between time points (Stark and Brown 2011). For each gene for each developmental stage, we also tallied the number of peaks between each gene and its up- and down-stream neighbor, as well as those within the introns of the gene, and the total height of the peak(s) overlapping the region directly adjacent to its transcription start site (TSS). For the spider, this was the start position ± 500 bp, and for the harvestman, this was the start position ± 300 bp, to scale for differences in genome size. Raw ATAC-seq reads were deposited in GenBank under the BioProject PRJNA1199734.

### Orthologue Definition

We used *OrthoFinder* (Emms and Kelly 2019) to define OGs in 11 available arachnid proteomes, using only the longest isoform for each gene (Table 1). Ohnologues were defined as described in the Results section.

**Table 1:** Species and genome accessions used in OrthoFinder

…	Species	Accession
WGD	*P. tepidariorum*	Science Data Bank doi:10.11922/sciencedb.o00019.00014
WGD	*A. bruennichi*	GenBank: GCA_947563725.1
WGD	*Stegodyphus dumicola*	GenBank: GCA_010614865.2
WGD	*Uloborus diversus*	GenBank: GCA_026930045.1
WGD	*D. silvatica*	https://github.com/molevol-ub/Dysdera_silvatica_genome
WGD	*Liocheles australasiae*	Wai Lok So and Jerome Hui, *personal comunication*
WGD	*Centruroides sculpturatus*	GenBank: GCA_000671375.2
…	*P. opilio*	This study: JBJXVG000000000
…	*Rhipicephalus sanguineus*	GenBank: GCA_013339695.2
…	*Dermacentor silvarum*	GenBank: GCA_013339745.2
…	*Ixodes scapularis*	GenBank: GCA_016920785.2

The left-most column indicates the species that share the arachnopulmonate WGD. The *P. opilio* genome can be accessed in our repository.

### Functional Annotation

To functionally characterize *P. opilio* and *P. tepidariorum* proteomes, we ran *InterProScan v5.70-102.0* and used the corresponding GO lookup table to find GO terms associated with each protein (Mitchell et al. 2015; Blum et al. 2021) for submission to *GO-Compass* (Harbig et al. 2023) for over-representation analysis.

## Supplementary Material

evaf238_Supplementary_Data

## Data Availability

The sequence data underlying this article are available in GenBank and can be accessed with the accession numbers cited in the Methods section, and processed data can be found on Figshare (https://doi.org/10.6084/m9.figshare.29655455). All code underlying this article is available on GitHub (https://github.com/madeleineaaseremedios/popt_code).
