# Supplementary figures and images for: Assessing the Impact of Whole Genome Duplication on Gene Expression and Regulation During Arachnid Development
Source: Genome Biol Evol. 2025 Dec 5;17(12):evaf238. doi: 10.1093/gbe/evaf238 (PMC12717030; doi:10.1093/gbe/evaf238)

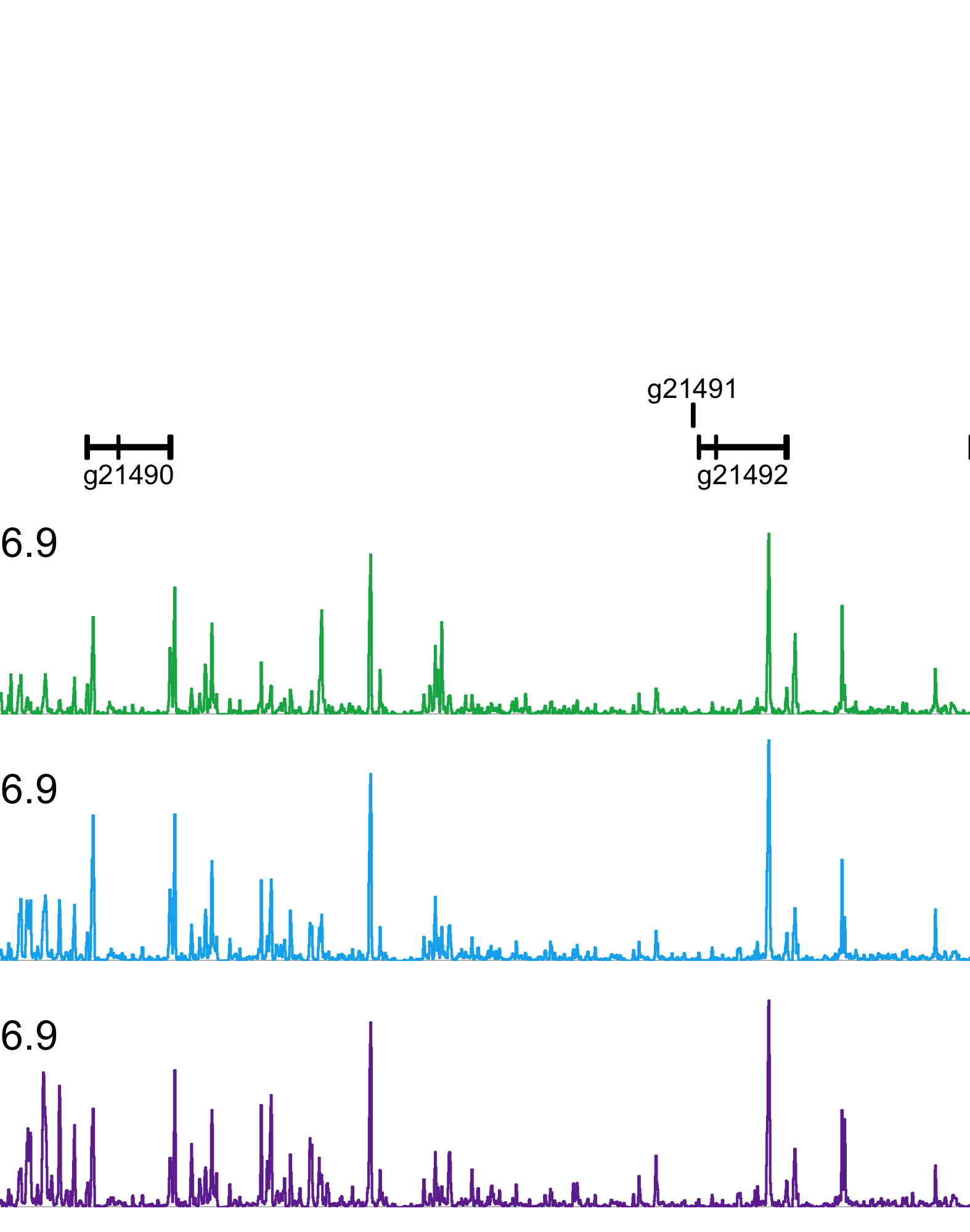

Supplement: evaf238_Supplementary_Data [file evaf238_supplementary_data.zip › supp-4/S3_hboxatacprofiles/po_emx1_emx2_pog21490-pog21492.png]

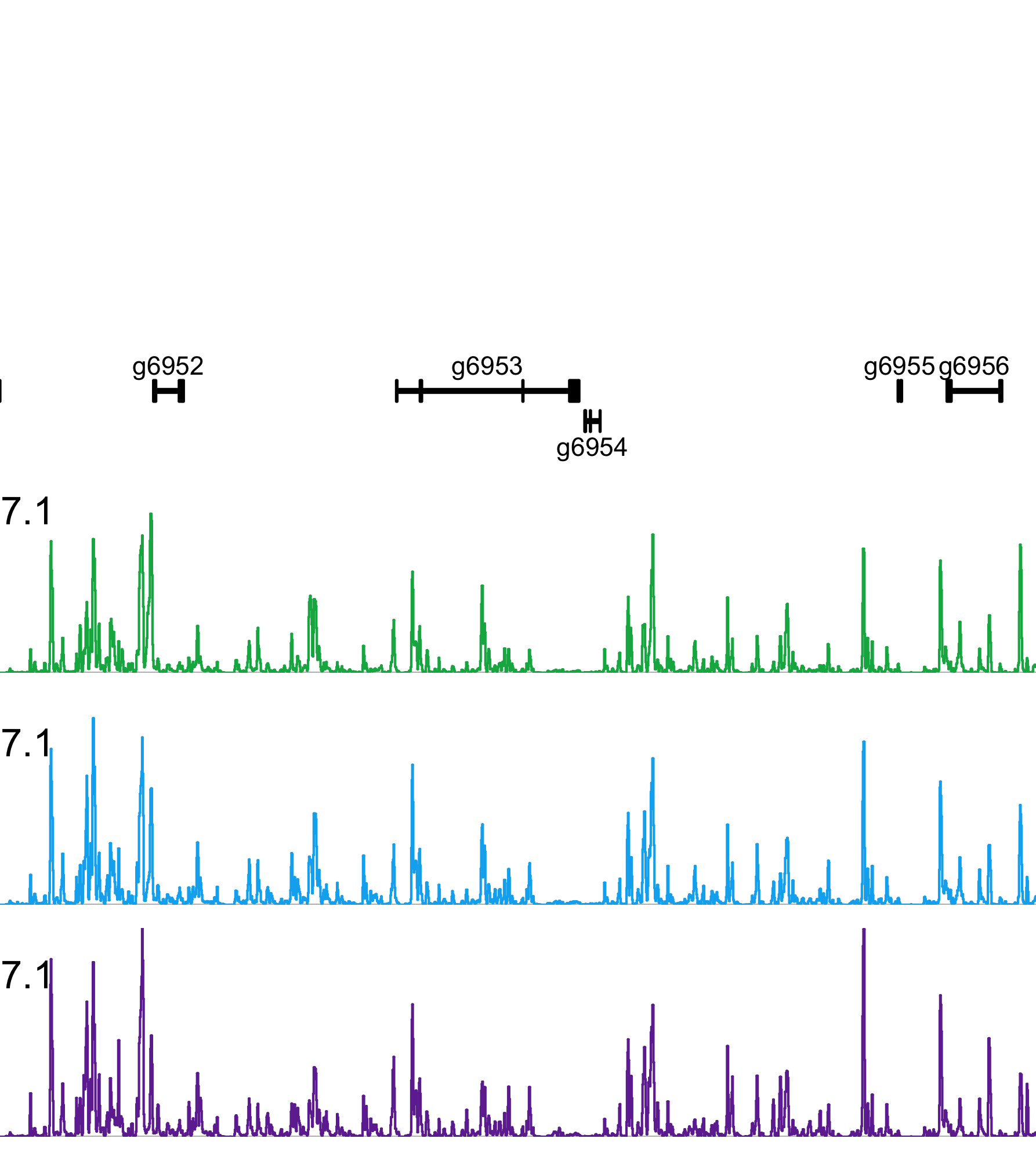

Supplement: evaf238_Supplementary_Data [file evaf238_supplementary_data.zip › supp-4/S3_hboxatacprofiles/po_hox_ant_pog6952-pog6956.png]

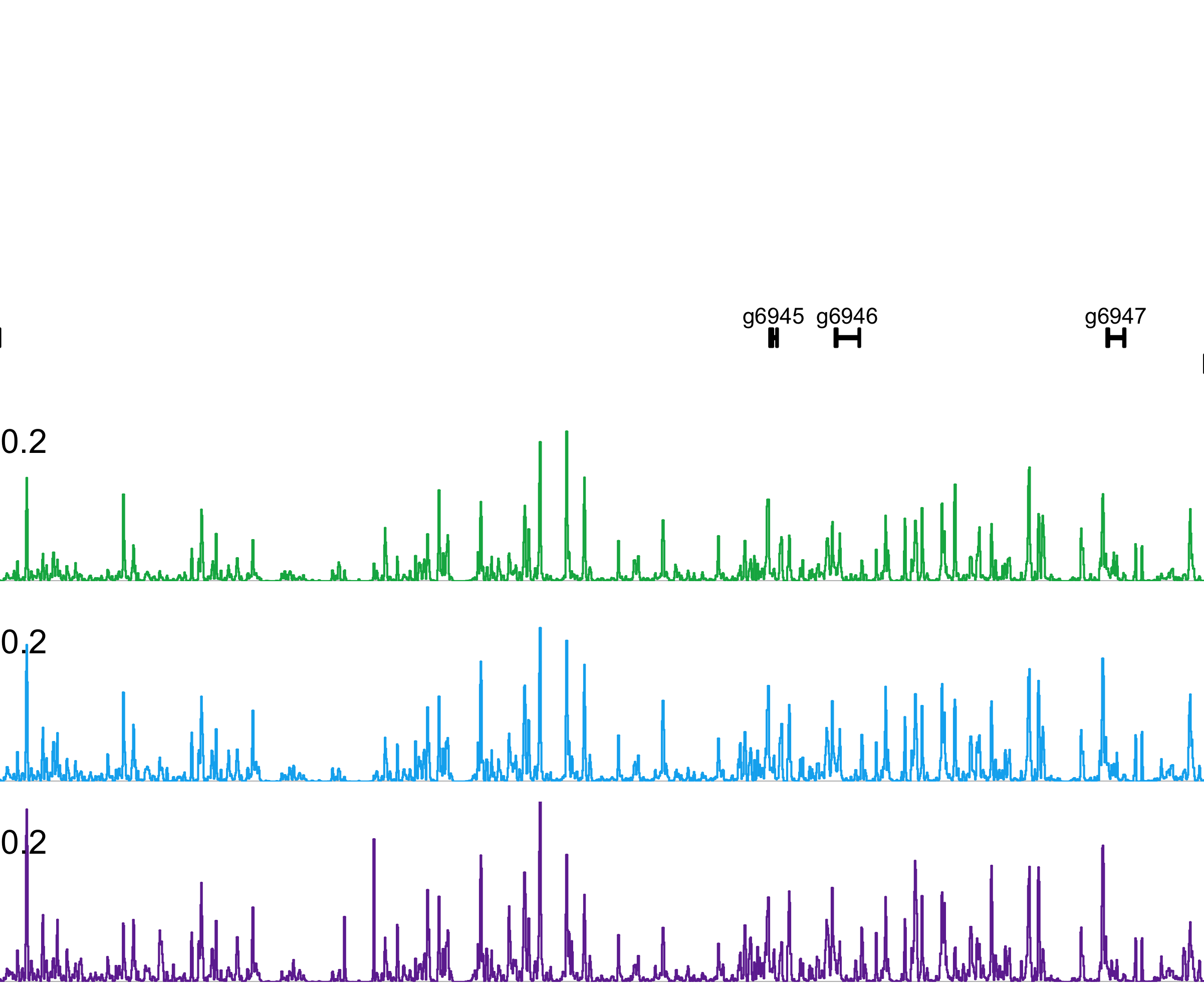

Supplement: evaf238_Supplementary_Data [file evaf238_supplementary_data.zip › supp-4/S3_hboxatacprofiles/po_hox_mid_pog6945-pog6947.png]

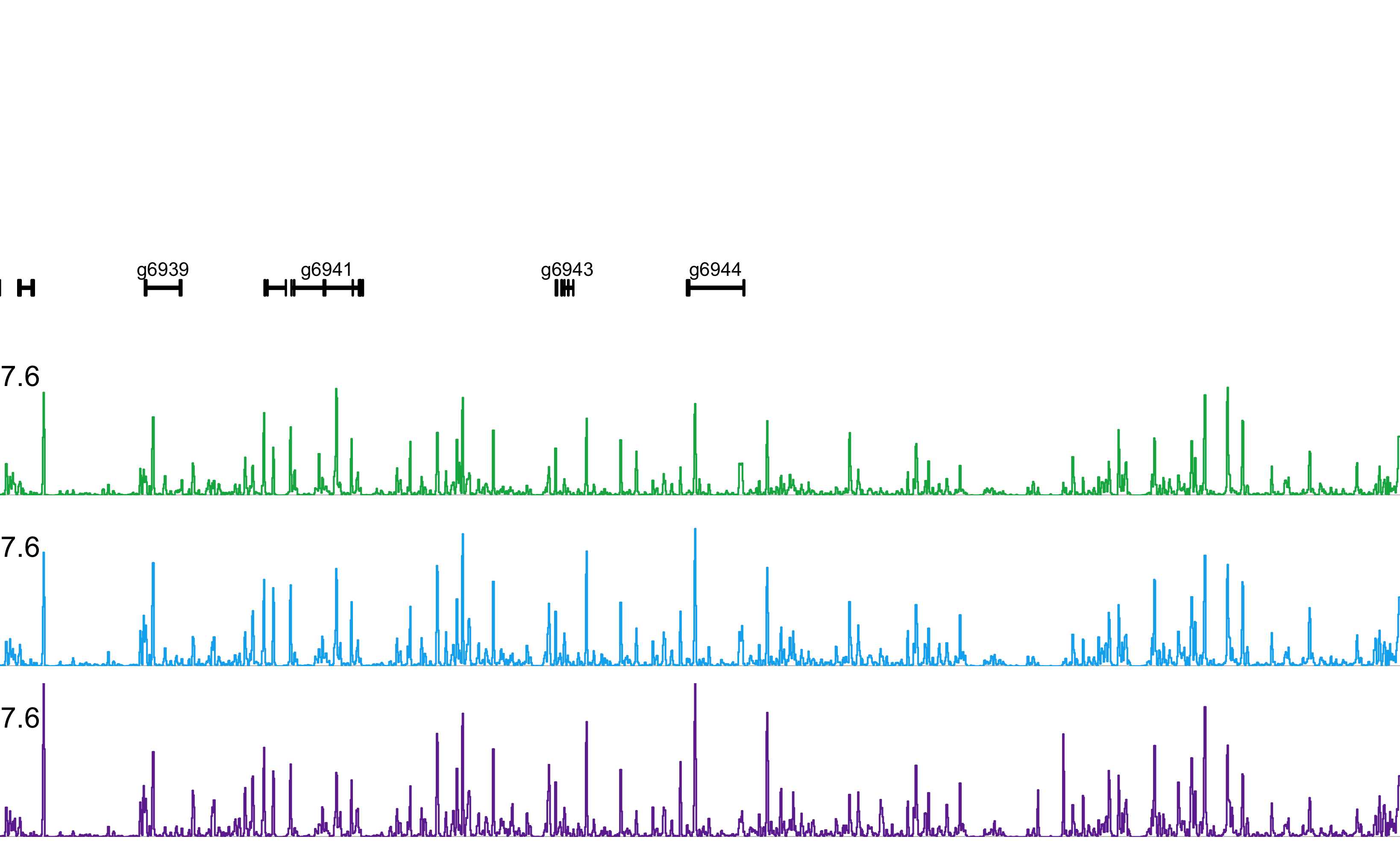

Supplement: evaf238_Supplementary_Data [file evaf238_supplementary_data.zip › supp-4/S3_hboxatacprofiles/po_hox_post_pog6938-pog6944.png]

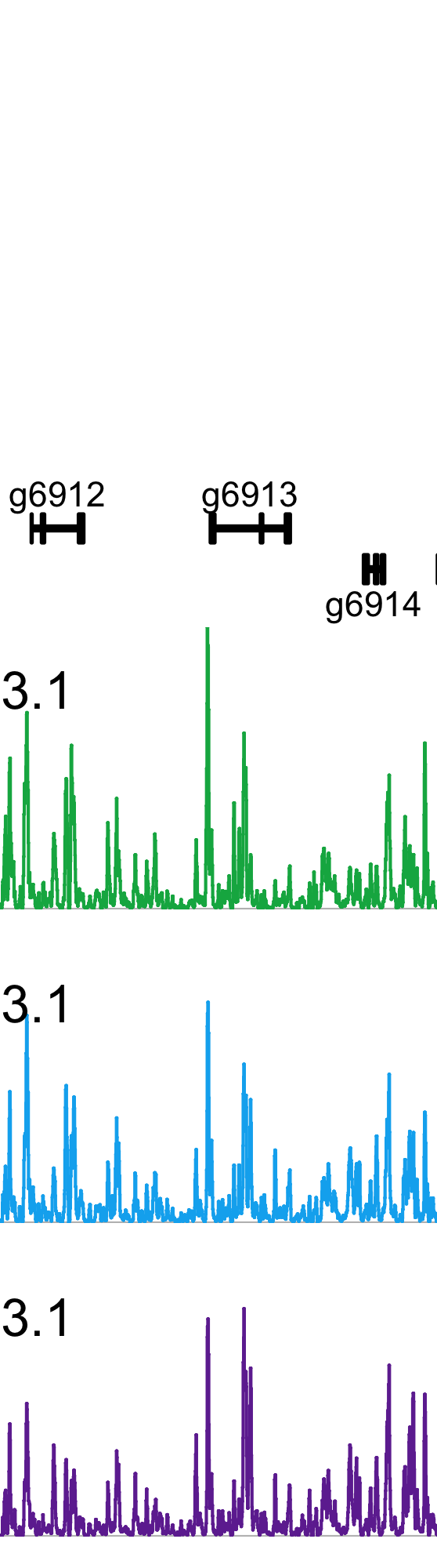

Supplement: evaf238_Supplementary_Data [file evaf238_supplementary_data.zip › supp-4/S3_hboxatacprofiles/po_hro_pog6912-pog6914.png]

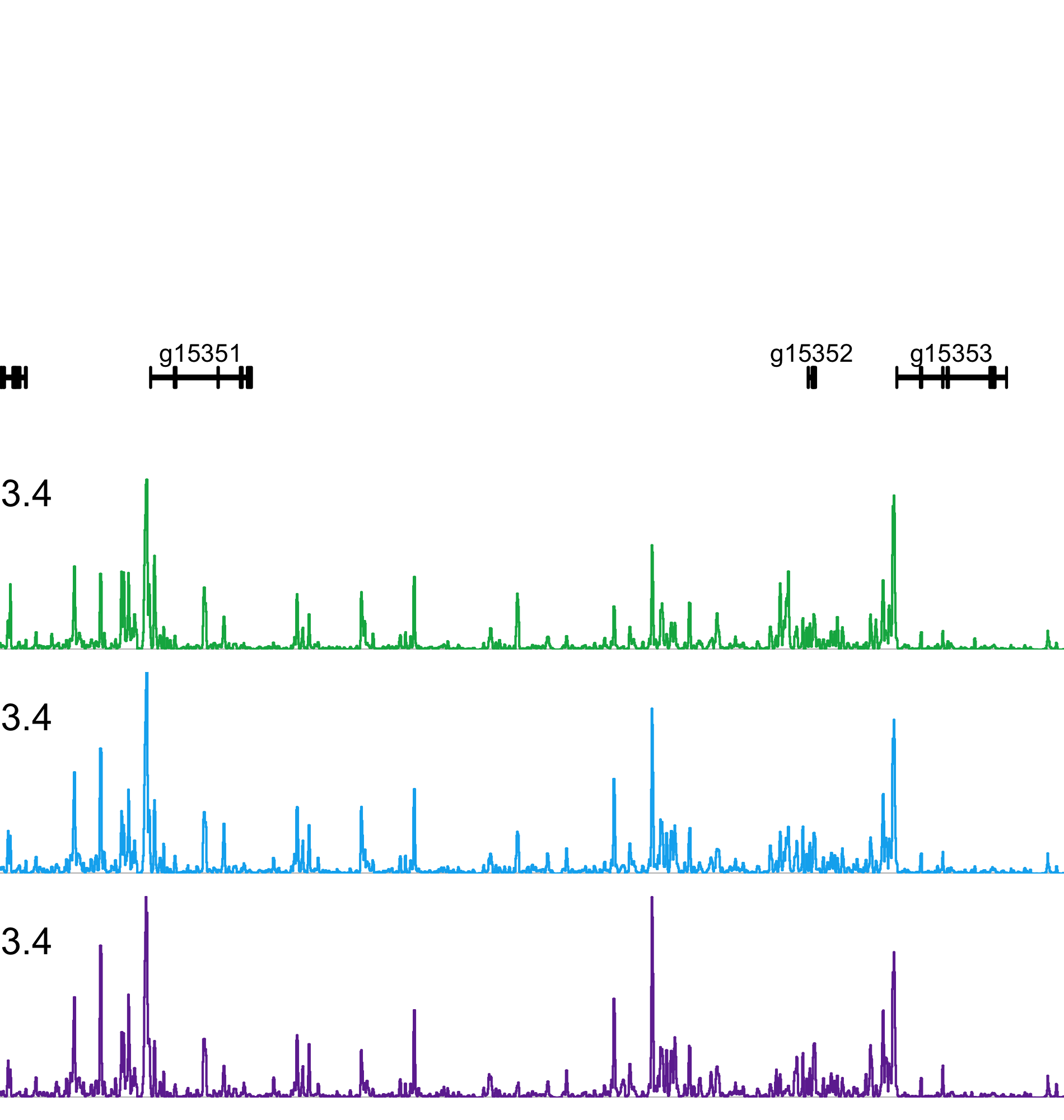

Supplement: evaf238_Supplementary_Data [file evaf238_supplementary_data.zip › supp-4/S3_hboxatacprofiles/po_irx_pog15350-pog15353.png]

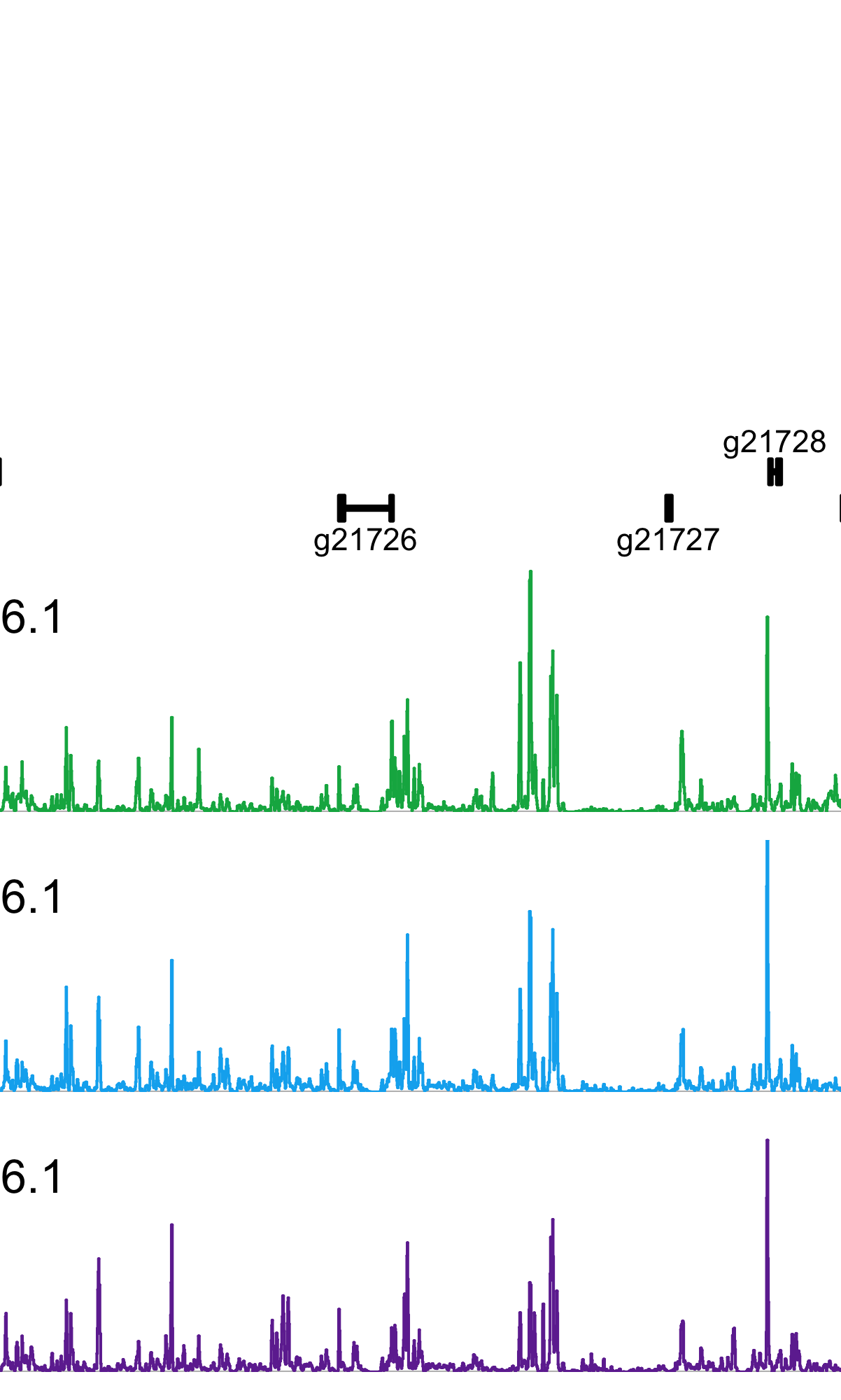

Supplement: evaf238_Supplementary_Data [file evaf238_supplementary_data.zip › supp-4/S3_hboxatacprofiles/po_nk5_nk1_pog21726-pog21728.png]

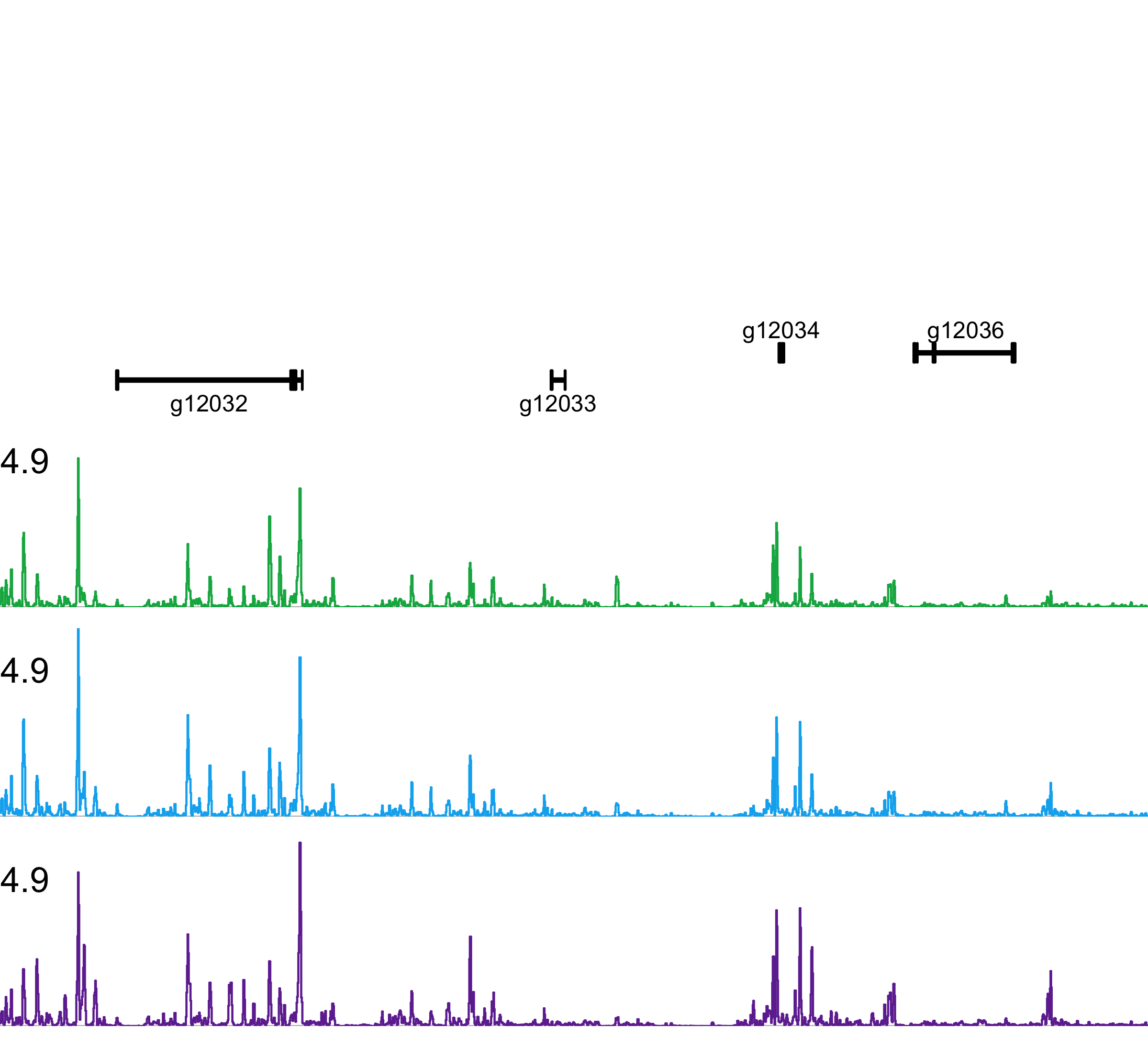

Supplement: evaf238_Supplementary_Data [file evaf238_supplementary_data.zip › supp-4/S3_hboxatacprofiles/po_nk7_lbx_pog12032-pog12036.png]

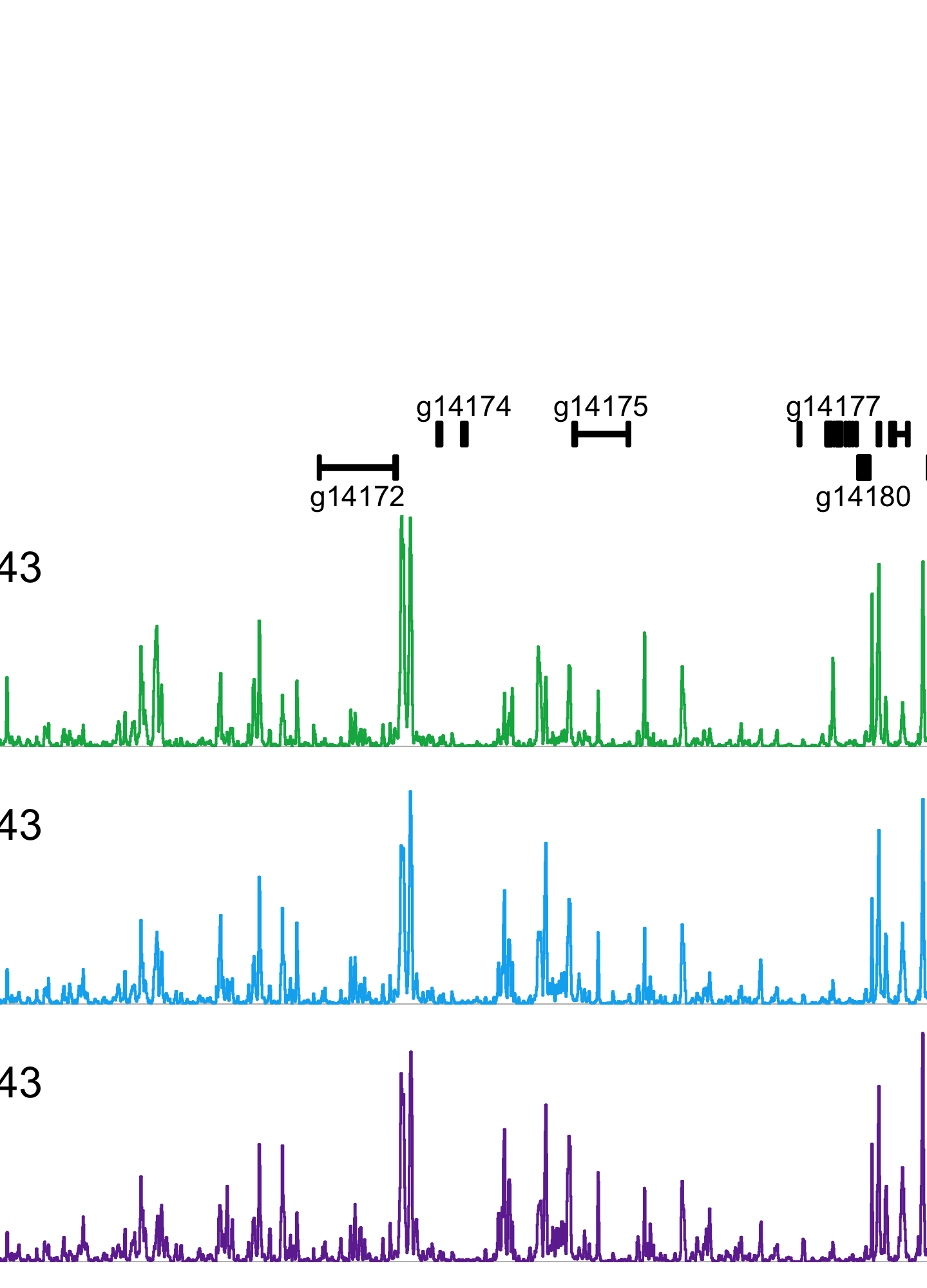

Supplement: evaf238_Supplementary_Data [file evaf238_supplementary_data.zip › supp-4/S3_hboxatacprofiles/po_six36_six45_pog14172-pog14182.png]

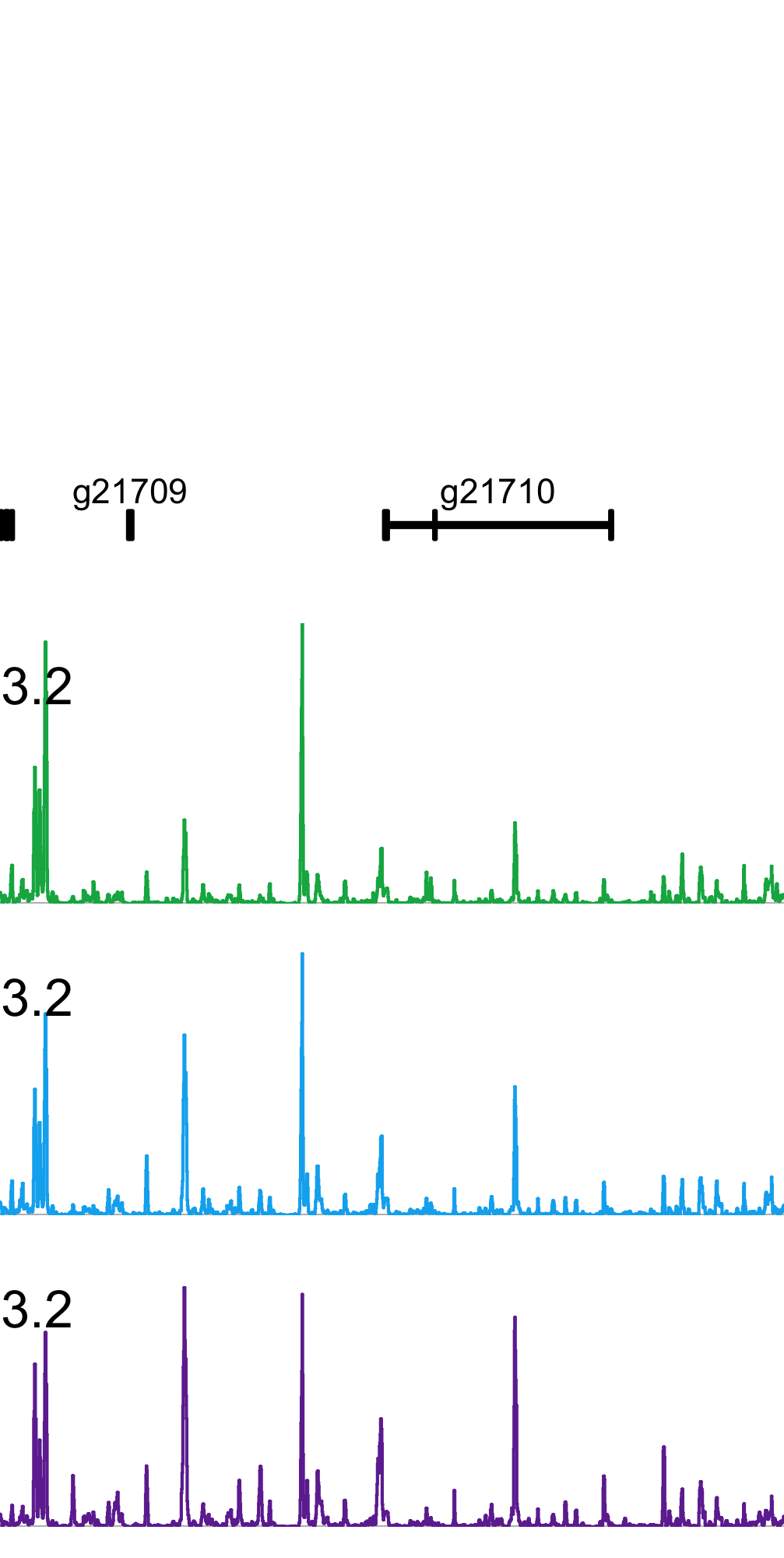

Supplement: evaf238_Supplementary_Data [file evaf238_supplementary_data.zip › supp-4/S3_hboxatacprofiles/po_tlx_nk3_pog21708-pog21710.png]

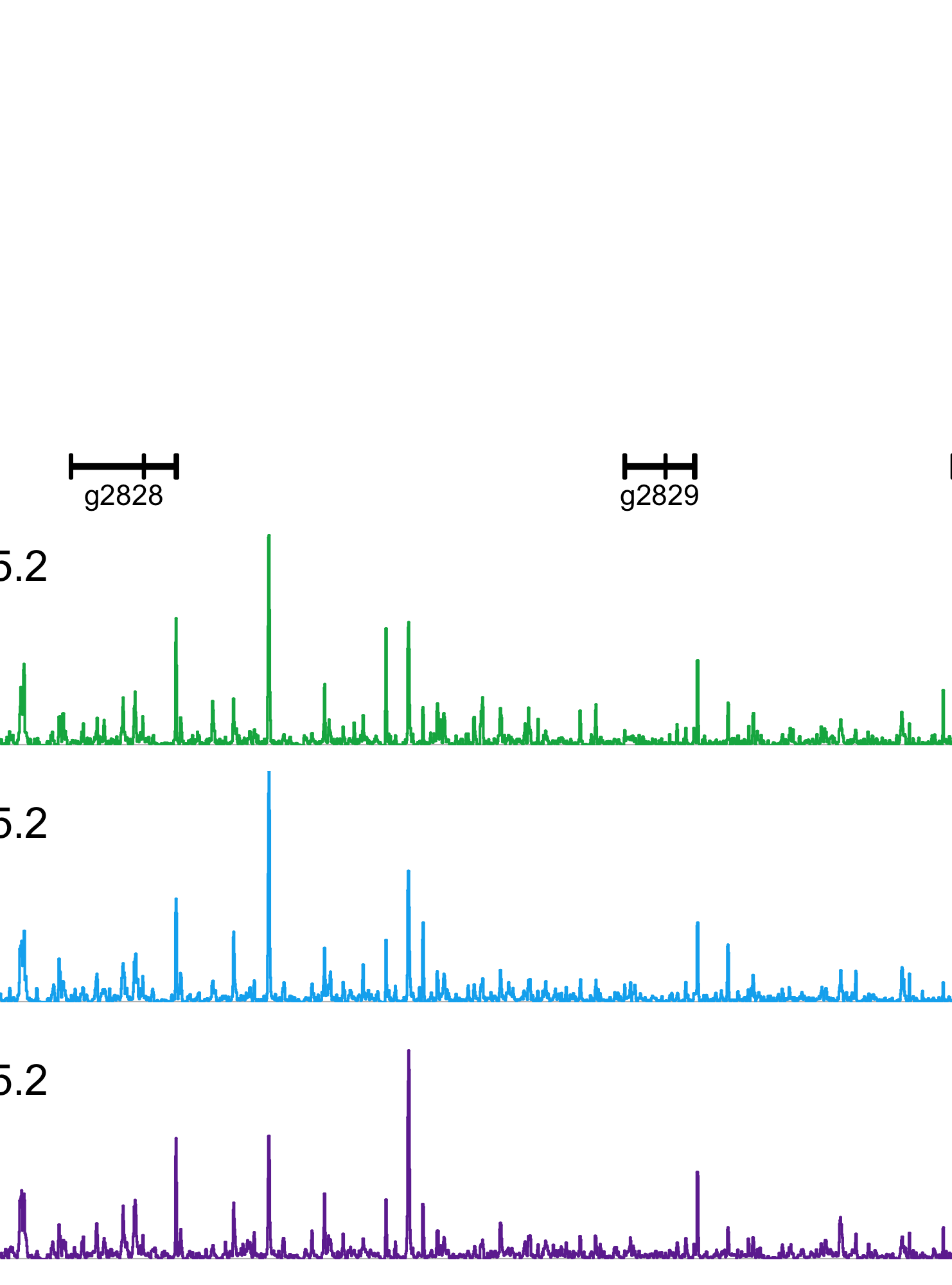

Supplement: evaf238_Supplementary_Data [file evaf238_supplementary_data.zip › supp-4/S3_hboxatacprofiles/ptD_emx1_emx2_ptg2828-ptg2829.png]

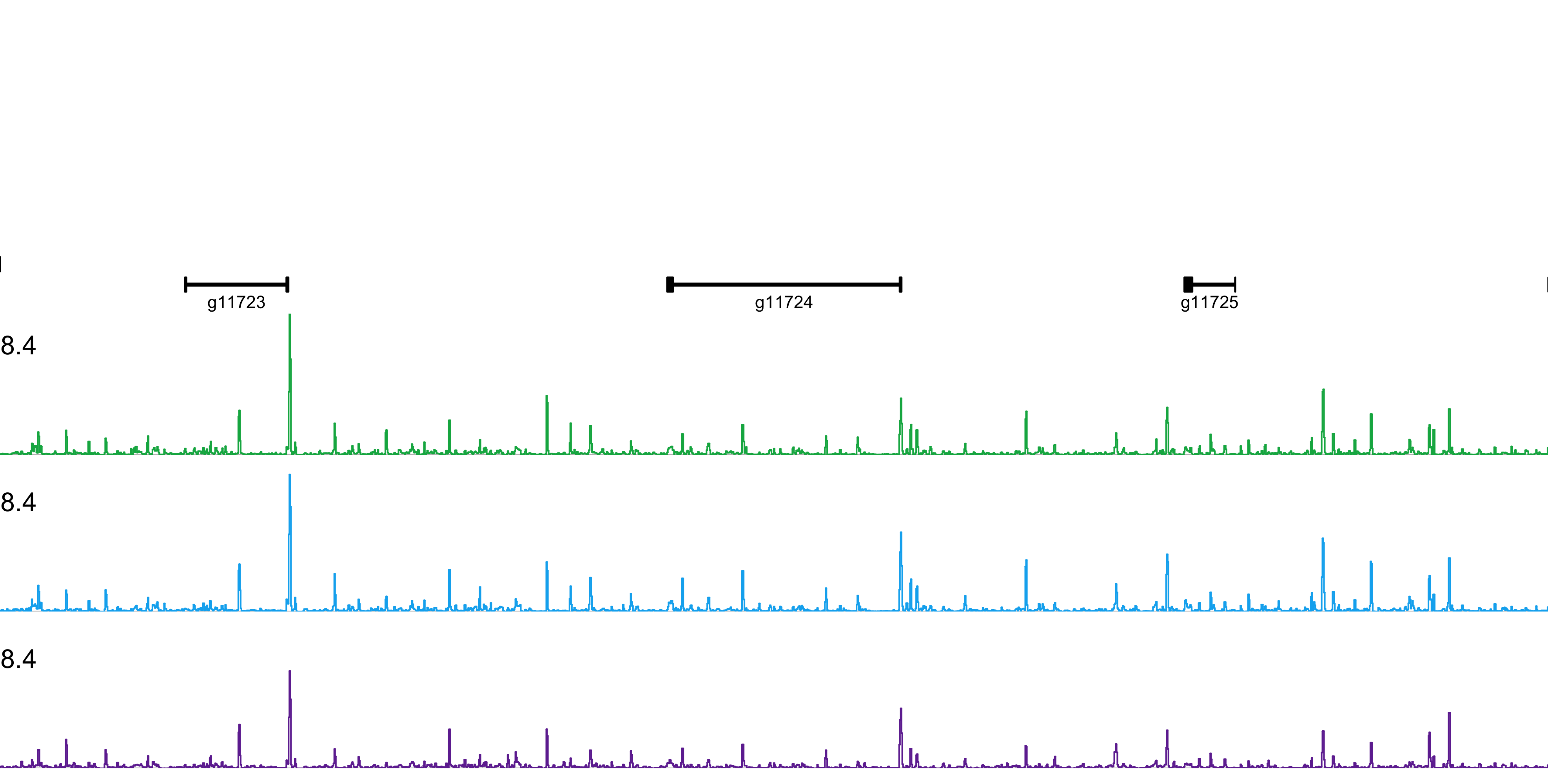

Supplement: evaf238_Supplementary_Data [file evaf238_supplementary_data.zip › supp-4/S3_hboxatacprofiles/ptD_hox_ant_ptg11723-ptg11725.png]

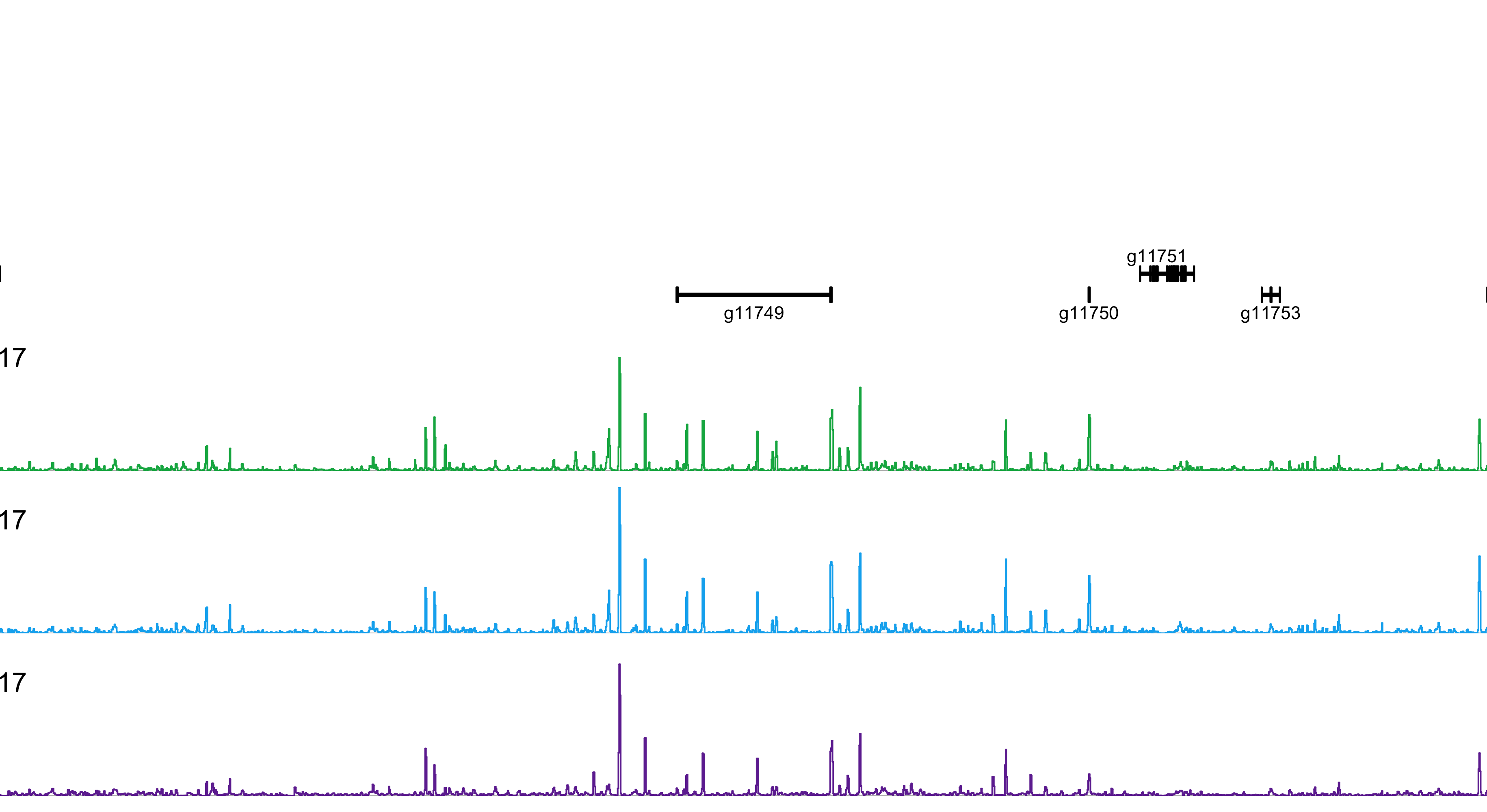

Supplement: evaf238_Supplementary_Data [file evaf238_supplementary_data.zip › supp-4/S3_hboxatacprofiles/ptD_hox_mid_ptg11749-ptg11753.png]

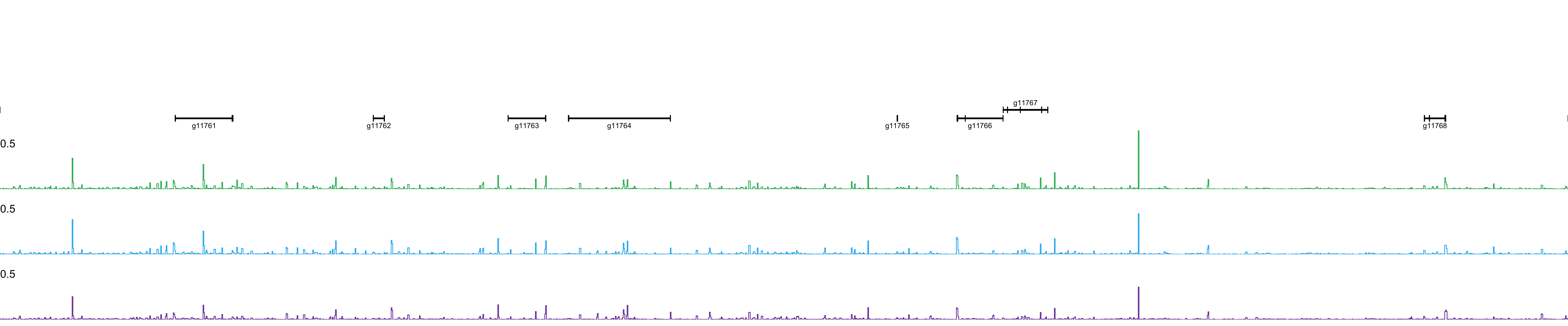

Supplement: evaf238_Supplementary_Data [file evaf238_supplementary_data.zip › supp-4/S3_hboxatacprofiles/ptD_hox_post_ptg11761-ptg11768.png]

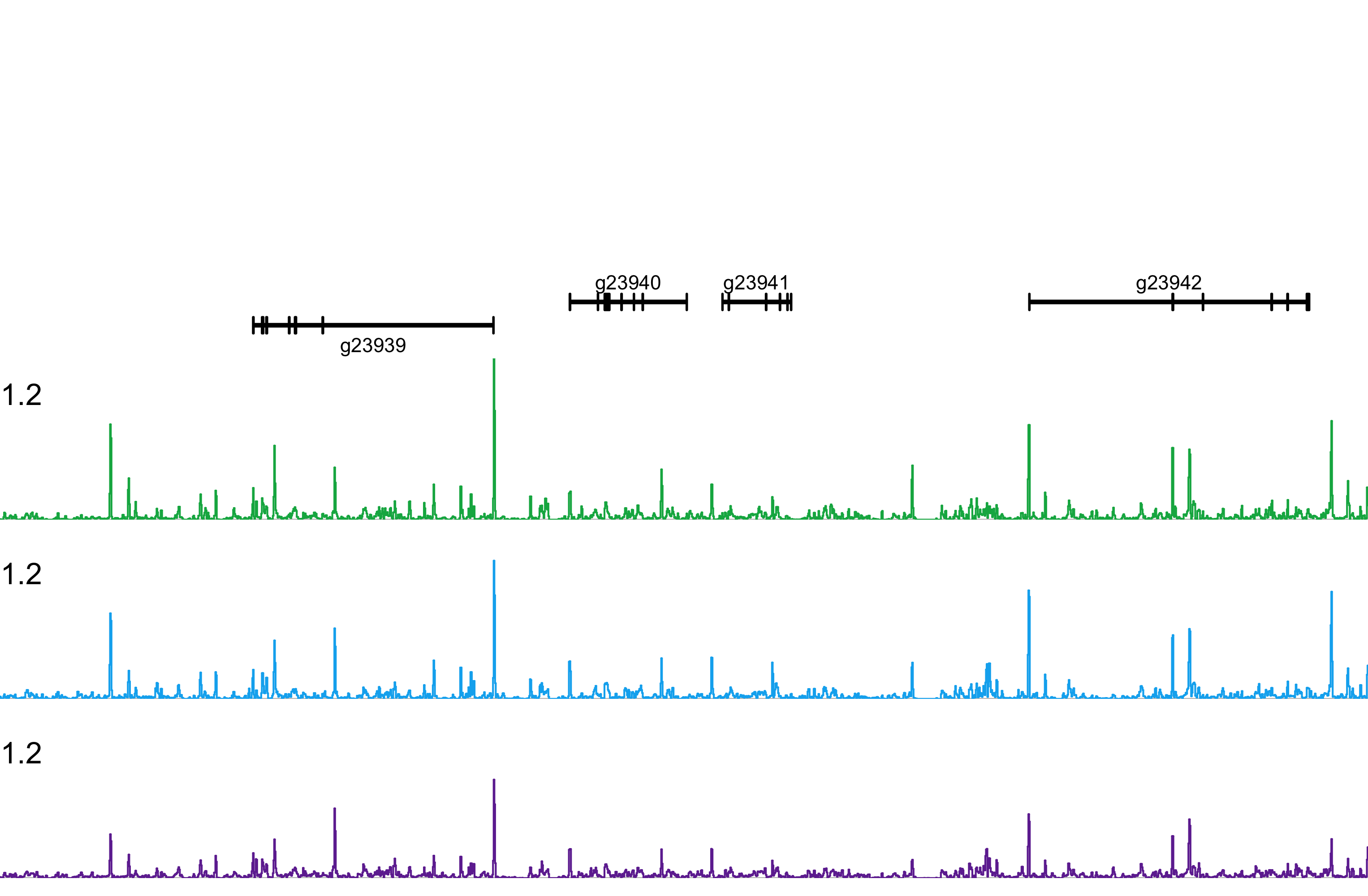

Supplement: evaf238_Supplementary_Data [file evaf238_supplementary_data.zip › supp-4/S3_hboxatacprofiles/ptD_irx_ptg23939-ptg23942.png]

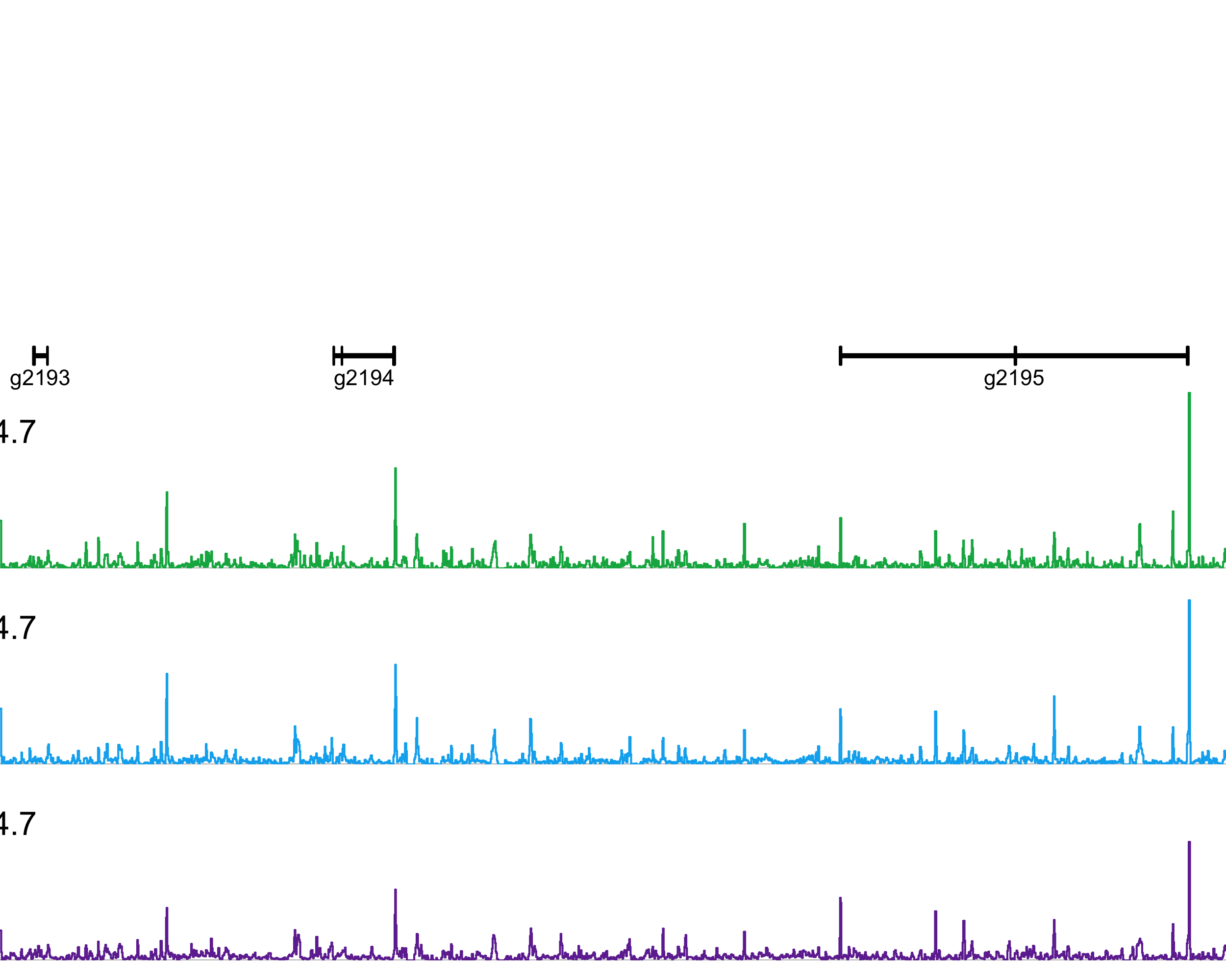

Supplement: evaf238_Supplementary_Data [file evaf238_supplementary_data.zip › supp-4/S3_hboxatacprofiles/ptD_lbx_tlx_ptg2193-ptg2195.png]

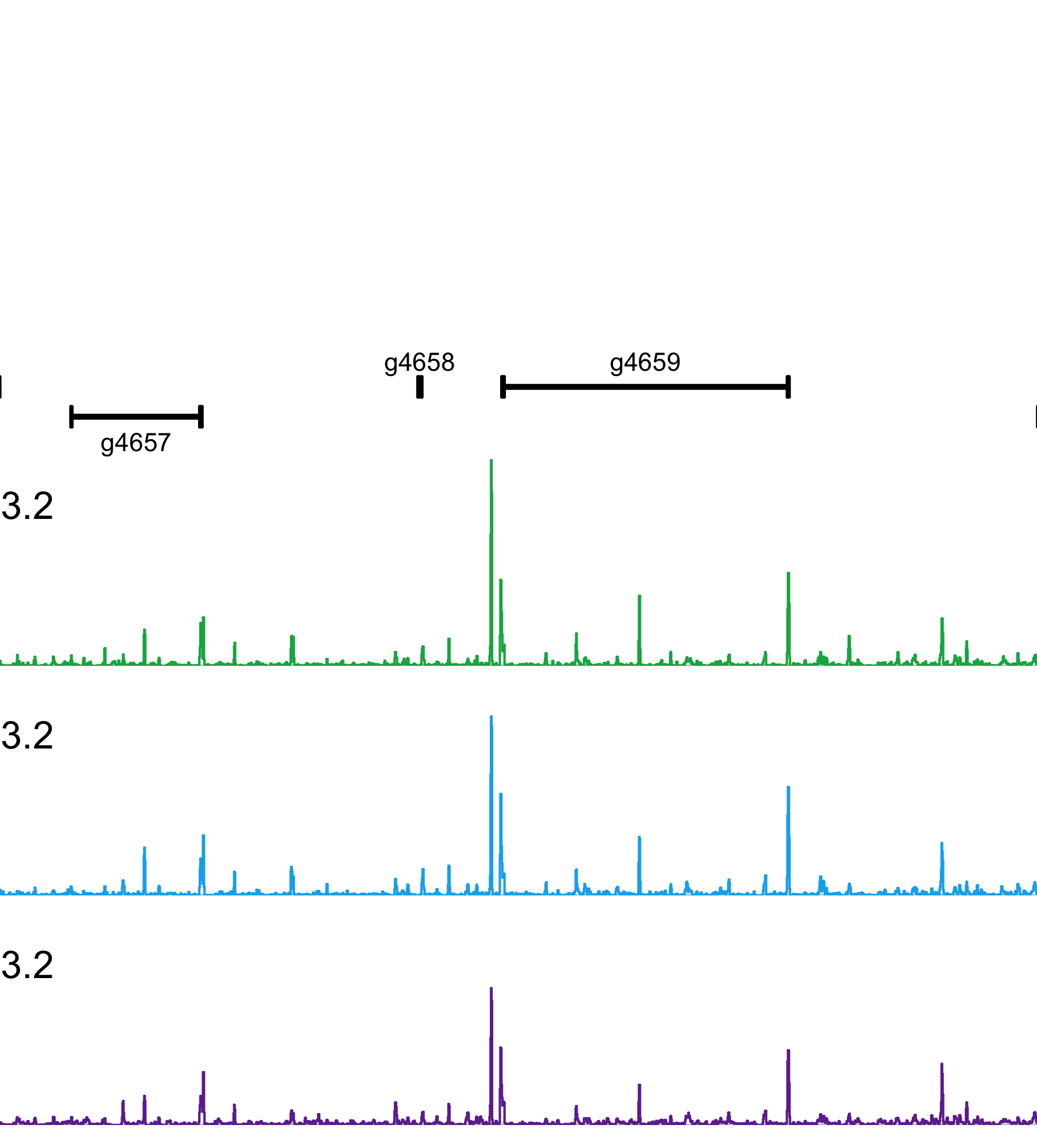

Supplement: evaf238_Supplementary_Data [file evaf238_supplementary_data.zip › supp-4/S3_hboxatacprofiles/ptD_six36_six12_ptg4657-ptg4659.png]

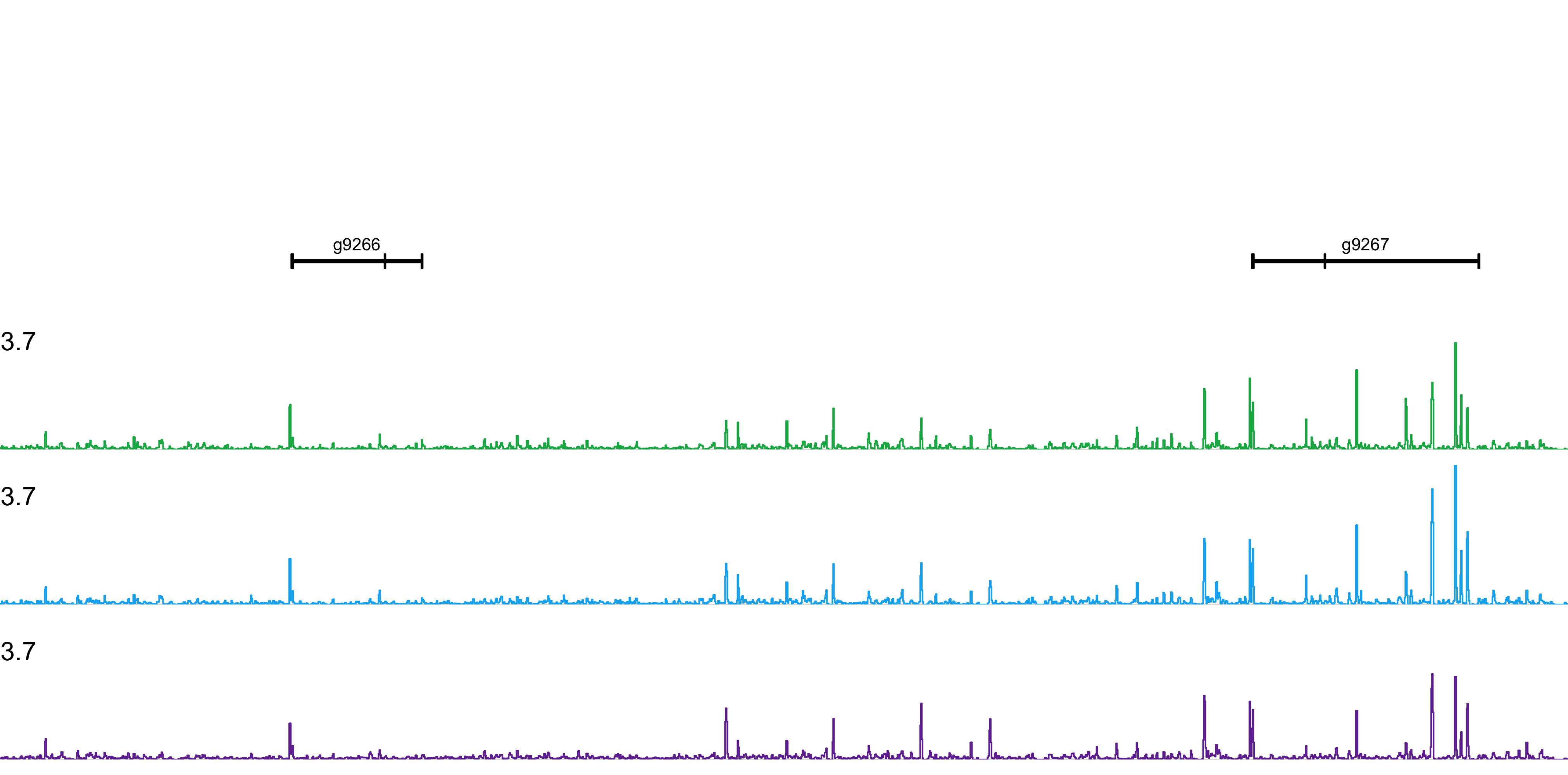

Supplement: evaf238_Supplementary_Data [file evaf238_supplementary_data.zip › supp-4/S3_hboxatacprofiles/ptI_emx1_emx2_ptg9266-ptg9267.png]

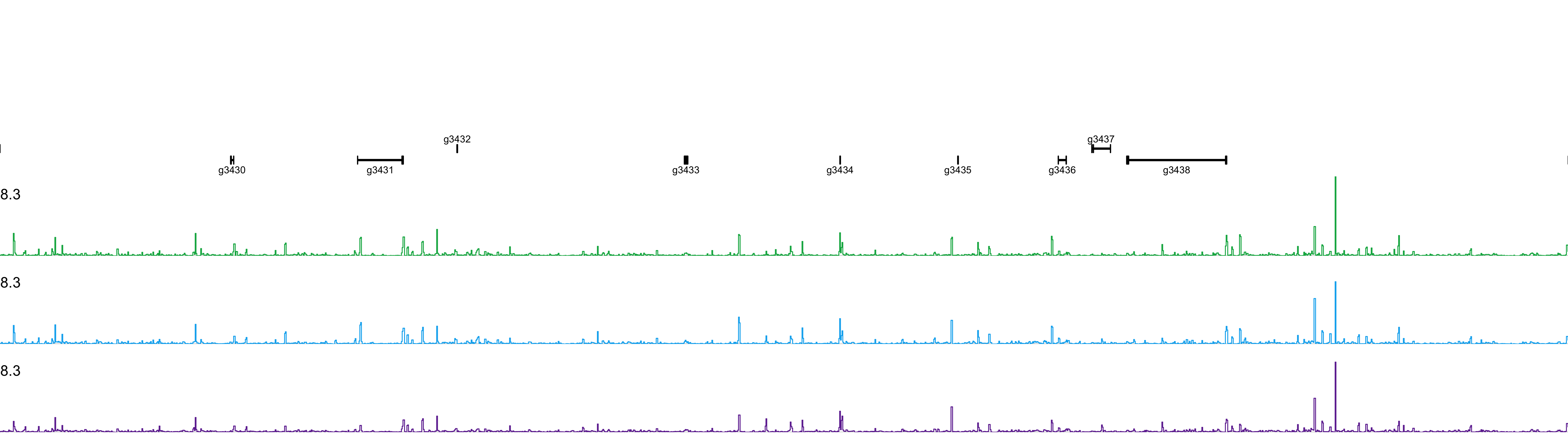

Supplement: evaf238_Supplementary_Data [file evaf238_supplementary_data.zip › supp-4/S3_hboxatacprofiles/ptI_hox_ant_ptg3430-ptg3438.png]

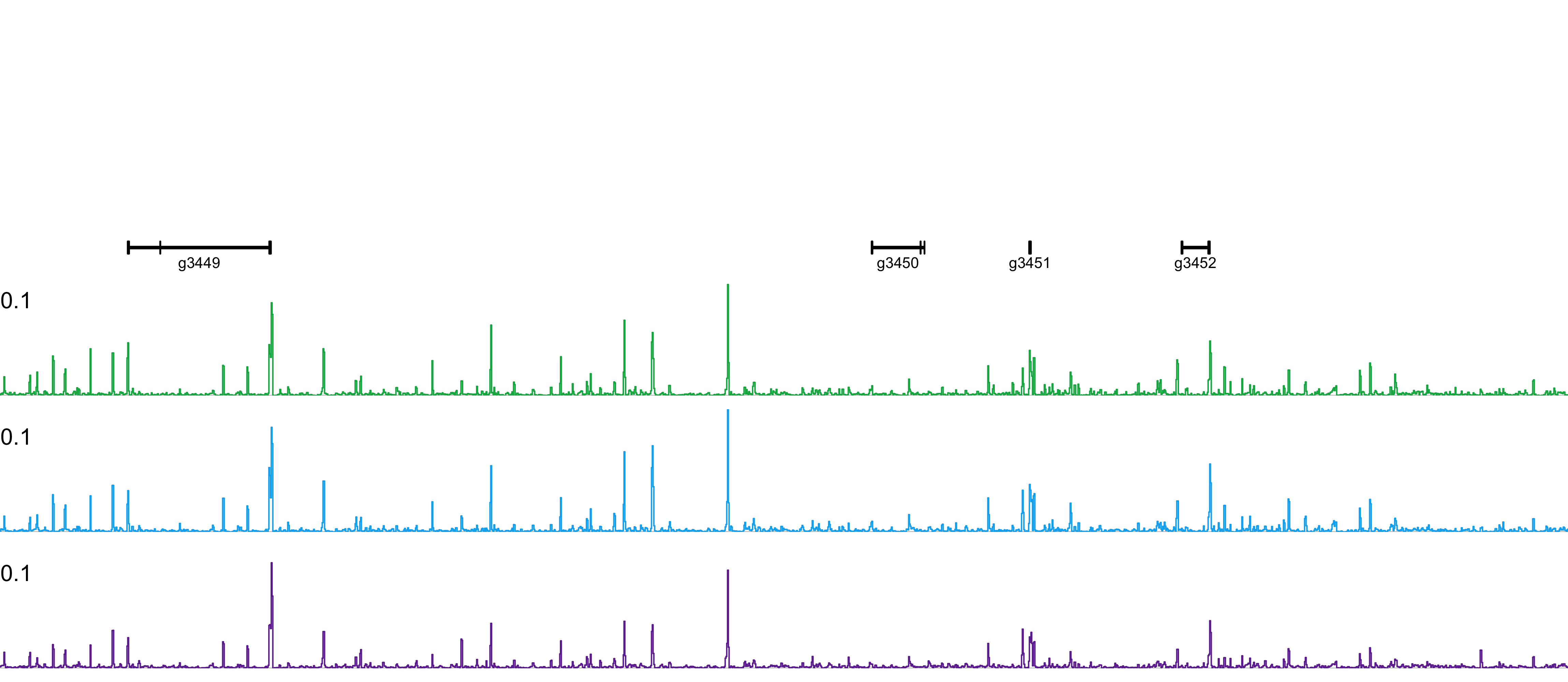

Supplement: evaf238_Supplementary_Data [file evaf238_supplementary_data.zip › supp-4/S3_hboxatacprofiles/ptI_hox_mid_ptg3449-ptg3452.png]

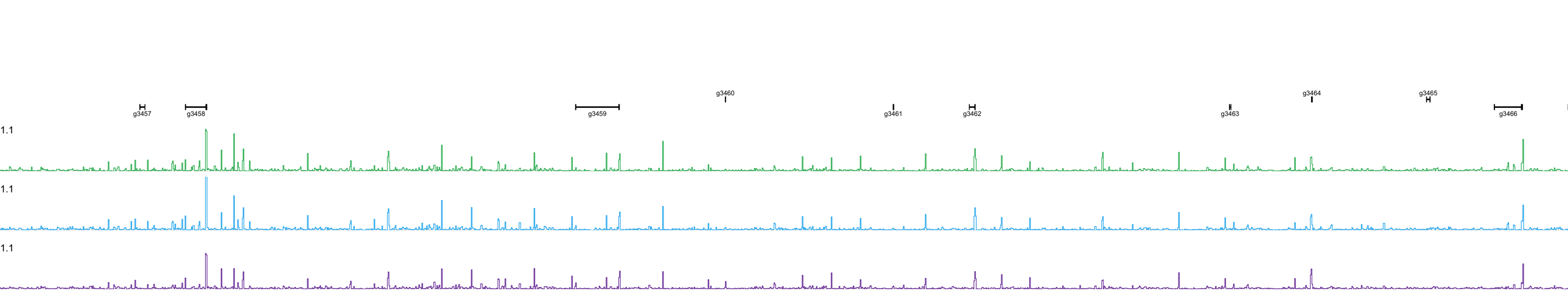

Supplement: evaf238_Supplementary_Data [file evaf238_supplementary_data.zip › supp-4/S3_hboxatacprofiles/ptI_hox_post_ptg3457-ptg3466.png]

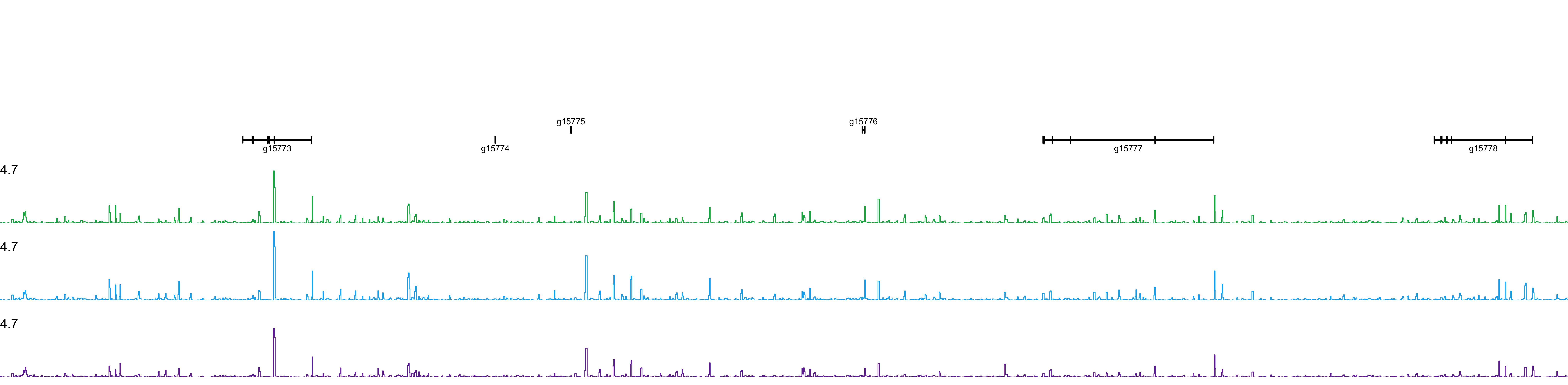

Supplement: evaf238_Supplementary_Data [file evaf238_supplementary_data.zip › supp-4/S3_hboxatacprofiles/ptI_irx_ptg15773-ptg15778.png]

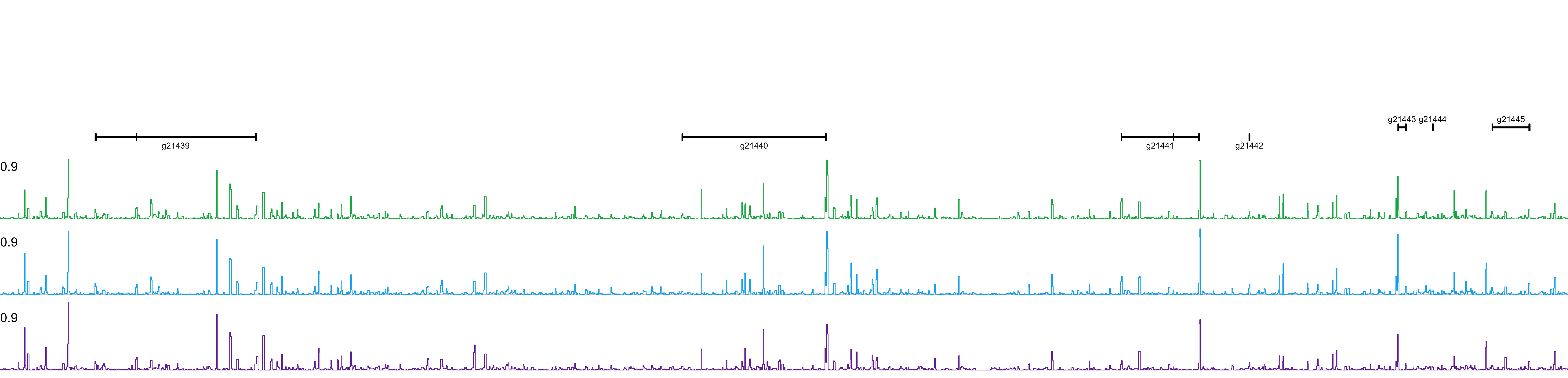

Supplement: evaf238_Supplementary_Data [file evaf238_supplementary_data.zip › supp-4/S3_hboxatacprofiles/ptI_nk_core_ptg21439-ptg21445.png]

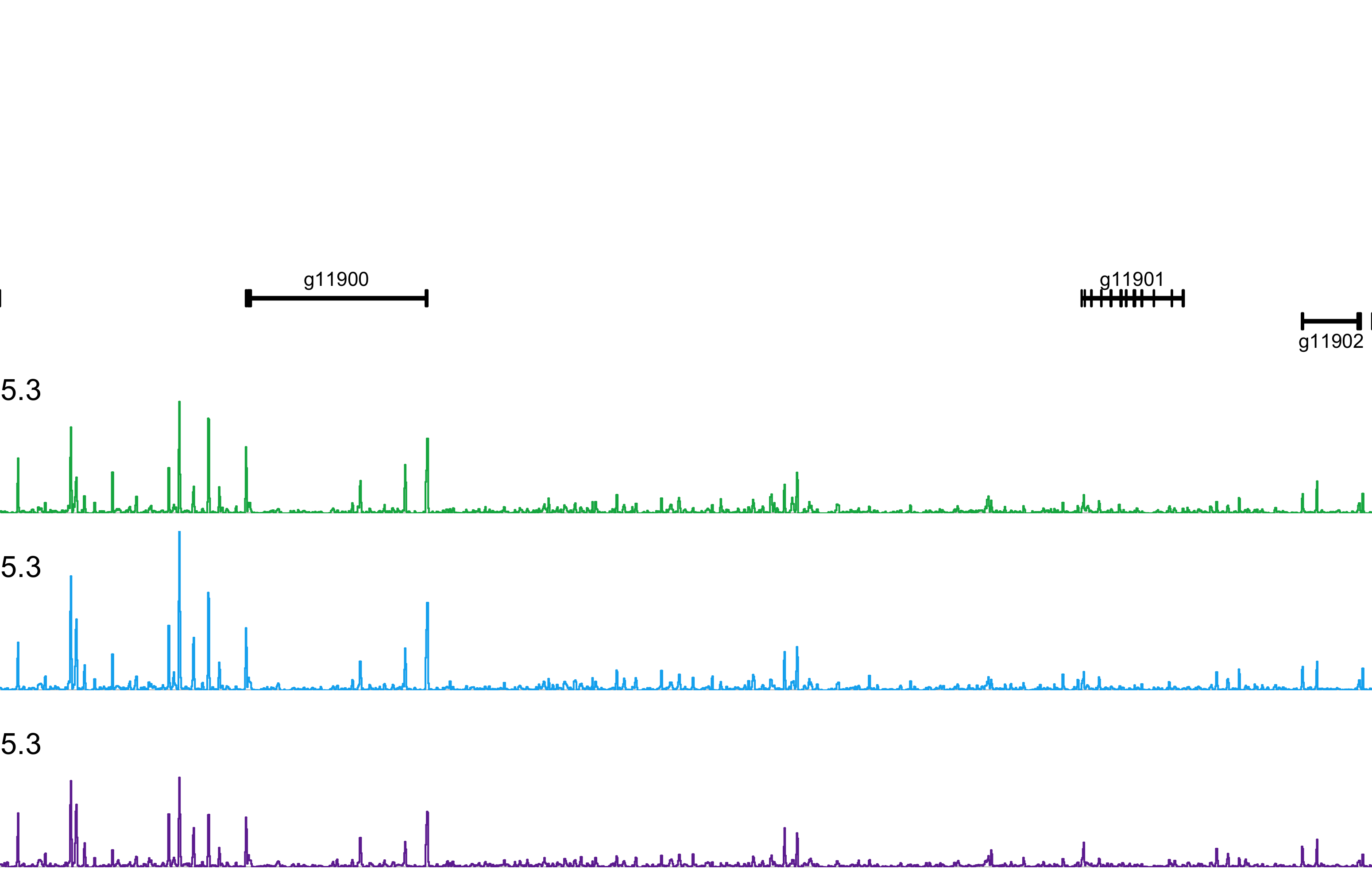

Supplement: evaf238_Supplementary_Data [file evaf238_supplementary_data.zip › supp-4/S3_hboxatacprofiles/ptI_six12_six45_ptg11900-ptg11902.png]

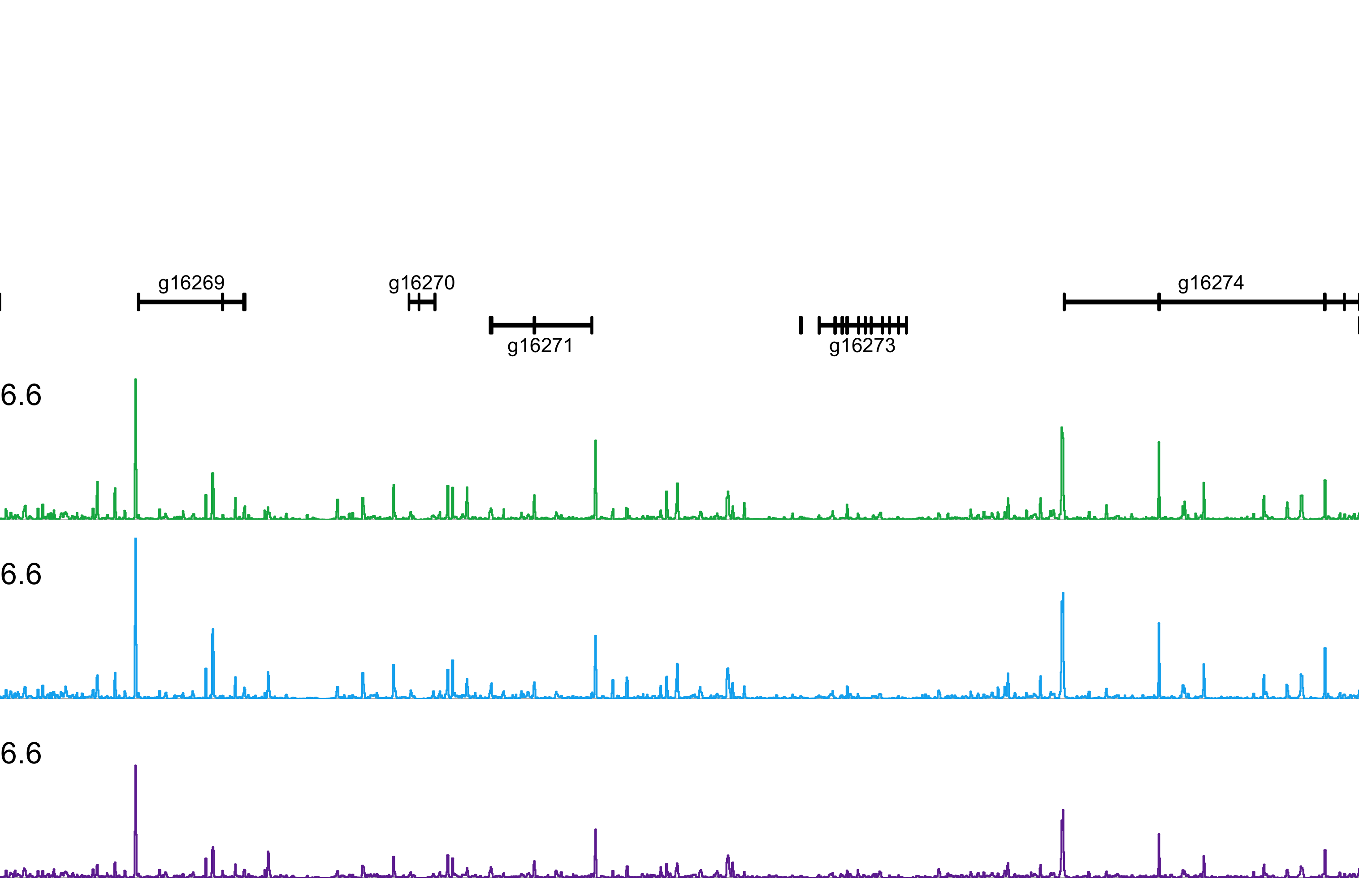

Supplement: evaf238_Supplementary_Data [file evaf238_supplementary_data.zip › supp-4/S3_hboxatacprofiles/pt_hro_ptg16269-ptg16274.png]

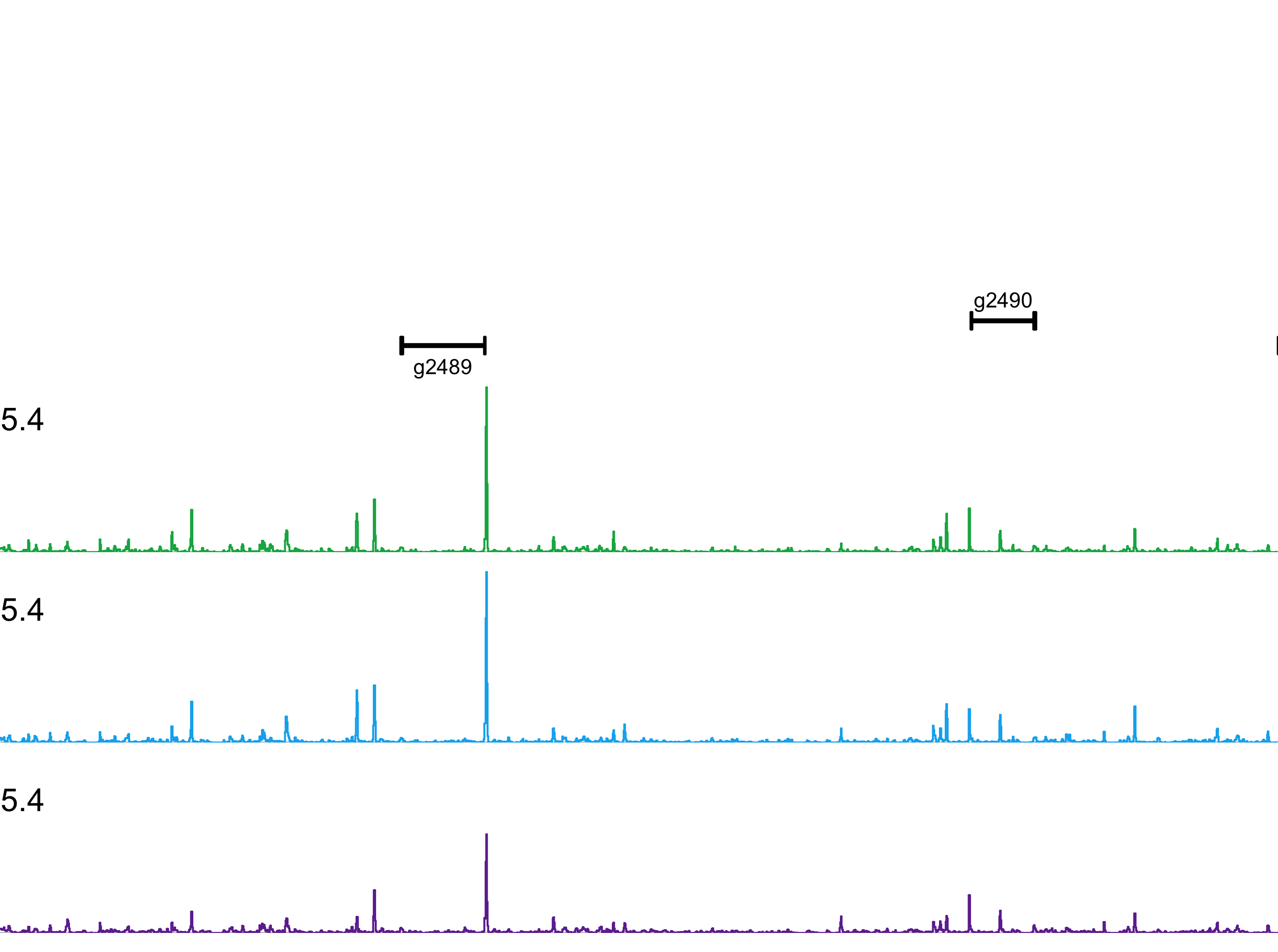

Supplement: evaf238_Supplementary_Data [file evaf238_supplementary_data.zip › supp-4/S3_hboxatacprofiles/pt_nk5_nk12_ptg2489-ptg2490.png]
